# Artificial Neural Network-Derived Unified Six-Dimensional Potential Energy Surface for Tetra Atomic Isomers of the Biogenic [H, C, N, O] System

**DOI:** 10.1021/acs.jctc.2c00915

**Published:** 2023-02-03

**Authors:** Fatemeh Arab, Fariba Nazari, Francesc Illas

**Affiliations:** † Department of Chemistry, Institute for Advanced Studies in Basic Sciences, Zanjan45137-66731, Iran; ‡ Center of Climate Change and Global Warming, Institute for Advanced Studies in Basic Sciences, Zanjan45137-66731, Iran; § Departament de Ciència de Materials i Química Física & Institut de Química Teòrica i Computacional (IQTCUB), Universitat de Barcelona, C/Martí i Franquès 1, 08028Barcelona, Spain

## Abstract

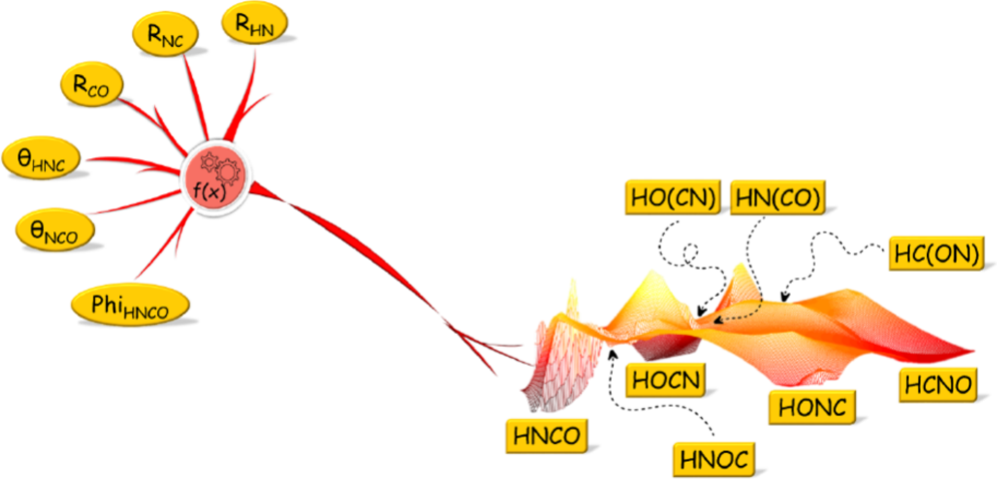

Recognition of different structural patterns in different potential energy surface regions, such as in isomerizing quasilinear tetra atomic molecules, is important for understanding the details of underlying physics and chemistry. In this respect, using three variants of artificial neural networks (ANNs), we investigated the six-dimensional (6-D) singlet potential energy surfaces (PES) of tetra atomic isomers of the biogenic [H, C, N, O] system. At first, we constructed a separate ANN potential for each of the studied isomers. In the next step, a comparative assessment of the separate ANN models led to the setting up of a unified 6-D singlet PES equally and accurately describing all studied isomers. The constructed unified model yields relative energies comparable to those obtained either from the gold standard CCSD(T) method or from separate ANNs for each of the studied isomers. The accuracy of the unified singlet PES is on the order of 10^–4^ Hartrees (0.1 kcal/mol). The developed PES in this work captures the main features of nonlinear and quasilinear tetra atomic isomers of this biogenic system.

## Introduction

To reach a deep scientific insight and also for technological improvement in many areas of physics, chemistry, and materials science, a quantitative description of atomic-scale phenomena is central. In this sense, ab initio methods provide accurate predictions of the properties of polyatomic systems, bulk crystals, and surfaces.^1^ Among the various calculated properties, the potential energy surface (PES) is of particular importance as it is the basis for subsequent studies involving the dynamics of a given system. A global PES is expected to reproduce experimental data at regions of the nuclear configuration space where such information is available and behave in a similar accurate manner elsewhere; namely, at intermediate and long-range regions far from the equilibrium structures.^2,3^ The actual knowledge of the chemical reactivity based on the complete construction of the PES represents a very important challenge in theoretical chemistry.^4^ Some of the major theoretical challenges found in building a PES from appropriate calculations are as follows: (i) determining a robust method for obtaining electronic energies that can be used in the construction of the PES; (ii) accurate fitting of the data without introducing spurious wiggles or unwanted details; (iii) correct consideration of symmetry; (iv) effective sampling and representing the most relevant regions of what is generally a vast configuration space especially when the number of atoms in the system is large; and (v) offering the possibility of a quick evaluation of the desired potential energy from the appropriate software interface for calling the PES when it is necessary.^2^

Currently, machine learning offers many opportunities to improve and efficiently obtain the PES of a given system.^5^ Many different methods have been proposed since the advent of the first machine learning potentials in the 1990s.^6,7^ For instance, Kocer et al.^8^ developed machine learning potentials based on ANNs. The current machine learning-based potentials include the early neural network potentials designed for low-dimensional systems,^9−15^ high-dimensional neural network potentials (HDNNPs) that take into account the environment-dependent atomic energy contributions,^16−25^ and fourth-generation HDNNPs, which include nonlocal phenomena such as long-range charge transfer.^26^ Furthermore, suitable, well-defined systems are necessary to evaluate the quality of the generated PES. Isomerization, one of the oldest and important significant concepts in chemistry, provides an ideal playground^27^ in this respect as it involves different equilibrium structures within a given PES.^28^

Global exploration of isomers on a quantum mechanical potential energy surface has been a long-standing problem since the 1960s,^29^ and quantum chemical methods are ideal for unraveling the isomerization reaction mechanisms and locating the PES-relevant stationary points, which, in the simplest case, involves two minima and one transition state.^30,31^

Precisely, isomerism and rich potential energy surfaces are unrivaled characteristics of the biogenic [H, C, N, O] system^32−37^ that provides an ideal candidate to evaluate the quality of PESs derived from different methods. Indeed, this is one of the most important and oldest systems related to PES investigation.^38^ The tetra atomic isomers of the biogenic [H, C, N, O] system have been extensively studied for a number of reasons.^39,40^ As an example, note that the peptide bond (N–C=O) plays a key role in metabolic processes since it links amino acids into peptide chains or proteins. Molecules containing peptide-like bonds have been detected across multiple environments in the interstellar medium, increasing the need to fully understand their chemistry and their role in forming larger prebiotic molecules.^41^ There exist intensive theoretical and experimental studies aimed at determining the PESs of tetra atomic isomers of the biogenic [H, C, N, O] system.^37,42−47^ However, in spite of various reports^48−52^ considering the PES of these tetra atomic isomers, there is not yet a comprehensive, coherent study providing a unified PES.

Based on a previous discussion, it appears that a suitable strategy to map PES with similar or better results than the traditional methods fitting ab initio data is the application of ANN potentials.^53^ Since ANN potentials have the largest diversity in terms of methods and concepts,^54,55^ we will here focus on the application of three variants of ANN potentials. These are those suggested by Brown and co-workers,^56^ Behler and Parrinello,^57^ and Zhang et al.^58^ We will make use of these ANNs to explore the six-dimensional singlet PES of the tetra atomic isomers of the biogenic [H, C, N, O] system with the aim of providing a unified potential energy surface model equally and accurately describing all studied isomers. Accordingly, we first revisit the potential energy surface of the most stable isomer HNCO and apply computational chemistry-based preprocessing to refine the data set. Next, we construct a separate PES model for each of the studied isomers and perform a comparative assessment of the three groups of ANN potentials. Finally, by combining the obtained individual PES models, we derive a unified PES model for the eight tetra atomic isomers of this system: namely, chain HCNO, HNCO, HOCN, HONC, HNOC, and several ring isomers. The presented unified PES is supposed to account for all underlying interactions^59^ and, by including all isomers, paves the road for the study of nuclear dynamics in this group of molecules.

### Computational Details

The construction of ANN potentials requires the compilation of reference data sets with atomic structures and their resultant accurate energies.^60^ Obviously, the quality of the obtained potential is sensitive to the data set used for training.^61^ The reference data set needs to not only use energy values as accurately as possible but also span the entire structural and chemical space that the ANN potential must represent for the intended application range and as few unnecessary or redundant data points as possible.^60^

To collect the data required to train the ANNs, we used the ORCA package version 3.0.3.^62^ First, the geometries of HCNO, HNCO, HOCN, HONC, and HNOC and a number of ring isomers were obtained from density functional theory-based calculations with the TPSS0 density functional^63^ and the def2-qzvpp basis set.^64^ To improve the accuracy of the required energy values, additional calculations were carried out for the density functional theory (DFT) optimized structures, as well as for a large set involving nearly 3000 generated structures, using the explicitly correlated, gold standard, coupled cluster method with single, double, and perturbative triple excitations (CCSD (T)) and also with the def2-qzvpp basis sets. The structure of the optimized tetra atomic isomers of the [H, C, N, O] system (S1–S8) is depicted in Figure S1 of the Supporting Information (SI) file.

**Figure 1:**
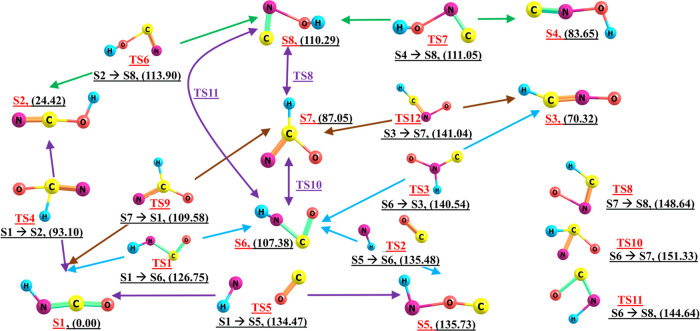
Isomerization paths and the involved transition-state structures in singlet PES. The S1–S8 and TS1–TS12 structures are representative of the stable isomers and transition states between each pair of isomers, respectively. Numbers in parentheses are relative energies in kcal/mol. Different paths are denoted by Sn → Sn, where *n* = 1–8 is the number of isomers.

All stationary points as well as minima and transition states have been characterized by computing harmonic vibrational frequencies via numerical Hessian built as finite differences from analytical gradients. The default convergence criteria in ORCA are used in geometry optimization and single-point energy calculations. In addition to the optimized structures, the relative energies, TS structures, and their paths are also reported in Figure 1.

### Constructing Artificial Neuronal Networks for [H, C, O, N] PES

To properly fit the PES, one needs to convert the information encoded in the molecular three-dimensional structure into an appropriate vector of numbers. This vector representation of a molecular structure is crucial in determining the success of the learning approach. Internal coordinate systems of representation have been proposed in the context of potential energy prediction.^65^ The set of collected data from ab initio calculations is then converted to a vector representation and used as input for the three variants of ANN as explained in detail below.

**Figure 2:**
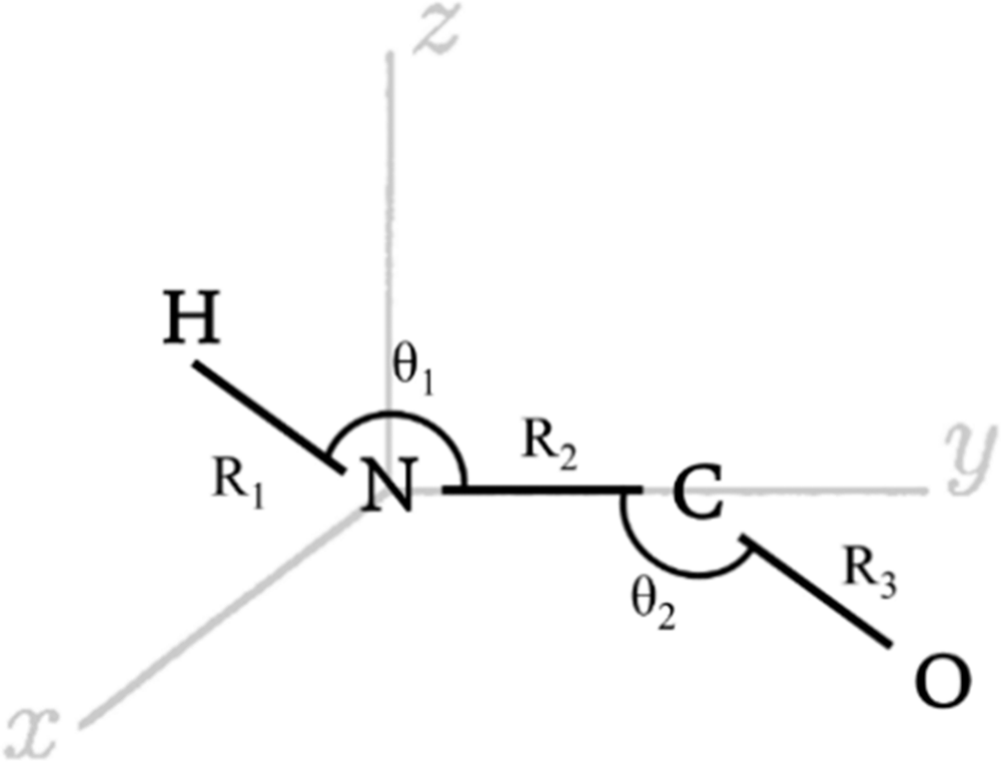
Valence body-fixed polyspherical coordinate system used for the HNCO molecule.

To generate the needed *XYZ* descriptors, six common internal coordinates for these eight optimized structures are defined. These are the N–H, C–N, and C–O distances that are designated as R_1_, R_2_, and R_3_, respectively, and the <HNC and <NCO angles, which are denoted as θ_1_ and θ_2_, respectively, and dihedral angle between HNC and NCO planes. To complete the description, a body-fixed polyspherical coordinate system is used for the tetra atomic isomers of the biogenic [H, N, C, O] system as shown in Figure 2. Additionally, to collect a separate data set for each of the structures shown in Figure S1, one-dimensional (1-D) to six-dimensional (6-D) regular mesh grids (RMGs) were generated along the physical coordinates centered at the equilibrium geometry. Further information regarding RMGs is summarized in Tables S1–S8 in the SI file. Note that RMGs that led to dissociation or nonphysical structures have been avoided using preprocessing techniques. We tried known methods like principal component analysis (PCA),^66^ the Kennard stone algorithm,^67^ and the energy filter.^68^ However, they showed no improvement in the performances of the trained neural networks. Consequently, to improve the performances and obtain consistent data for all studied isomers, we used Mayer bond^69^ analyses using a simple python code, one-dimensional PES of the structures, and special transition-state structures as a preprocessing technique. Accordingly, for each structure in Figure S1, we generated a total of 3000 data points and the energies of this structures were obtained at the CCSD(T)/def2-qzvpp level of theory. To construct the PES model, we adopted a molecular single-layer neural network (SLNN),^56^ a descriptor-based model atomic neural network (BPNN),^57^ and the graph convolutional atomic neural network (PiNet)^58^ as described below.

#### Details Regarding Obtaining the Single-Layer Neural Networks (SLNNs)

The simplest neural network is the SLNN introduced by Pradhan et al.^56^ for tetratomic molecules. Here, a training set is employed to fit the PES that contains 80% of the total data, including random geometries plus the 1-D RMGs. Next, 10% of the data points are used for validation and the test set so as to avoid overfitting of the training set and to examine the quality of the fit at the end of the fitting process, respectively. The Levenberg–Marquardt algorithm was used to determine the fitting parameters following eq 1. In this model, an exponential activation function is used that allows one to write the PES function in the form of sum-of-products^70^
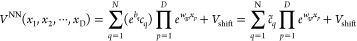
1and, before fitting, all data (coordinates and energies) are scaled as in eq 2
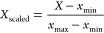
2where the maximum and minimum of a particular coordinate (or the energy) are *x*_max_ and *x*_min_, respectively, where *X* stands for values before scaling, which after scaling appears as *X*_scaled_. This ensures that the scaled data is in the [−1, 1] interval, which facilitates a smooth convergence and a gradual decrease in the root-mean-squared error (RMSE) for the fit. To find the best training set for SLNN, we randomly constructed five training, test, and validation sets. Next, a one-stage fitting procedure in a loop over five iterations was applied to reduce the RMSE. By comparing the RMSE of the training, test, and validation sets, the ones with minimum RMSE were chosen to optimize the number of neurons.

#### Details Regarding Obtaining the Descriptor-Based Neural Network (BPNNs)

The second type of neural network explored is the BPNN introduced by Behler and Parrinello.^57^ The BPNNs have more complexity than the SLNNs since the chemical environment around a given atom is represented by a rotationally, translationally, and permutationally invariant vector of symmetry function and atom center symmetry functions capture both radial and angular features of the chemical environment within a cutoff radius *R*_c_. Consequently, symmetry functions of each chemical element and its neural network architecture and the fitted parameters are a unique set for each atom. Here, we have utilized the atom-centered symmetry functions suggested by Behler and Parrinello^57^ as implemented in the PiNN package.^58^ In this scheme, the total energy of a system is decomposed into a sum of the contributions of all effective atomic energies *E_i_* from each atom
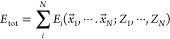
3where *x⃗_i_* is the atomic position and *Z_i_* is the atomic number. The calculation of effective atomic energies *E_i_* will be described later in this section. At this moment, it is sufficient to mention that, once computed in the atomic ANN, these are introduced to the designed ANN and the output of this ANN provides the total energy of the molecule. Summation of the effective atomic energies *E_i_* is done by equal weight in this work.

Using a set of Gaussian descriptor functions, the contribution to the total energy of each atom is computed. Note that the definition of the Gaussian descriptor is unique for each system and an important ingredient. Hence, using the NN designated by Behler, the atomic effective energy is calculated, and further details can be obtained in the original reference.^57^ The particular Gaussian descriptor functions used in the present work are as in eqs 4–6
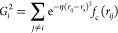
4
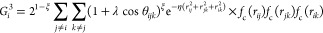
5
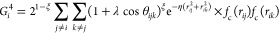
6where
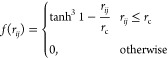
7The Gaussian symmetry function *G*_*i*_^2^ (eq 4) captures the interactions of an atom *i* with all atoms *j* within a cutoff distance, *R*_c_, whereas *G*_*i*_^3^ and *G*_*i*_^4^ symmetry functions (eqs 5 and 6) capture the three-body interactions between *i* and all *j* and *k* atoms for which the atomic separations *r_ij_*, *r_ik_*, and *r_jk_* are less than the cutoff distance and θ*_ijk_* is the angle formed by *r_ij_* and *r_ik_*. These functions, which encode the atomic position as well as the local neighborhood of each atom, are invariant to rotations and translations. In this way, the information is passed to a fully connected feed for the neural network. Here, *G*_*i*_^2^, *G*_*i*_^3^, and *G*_*i*_^4^ versions of symmetry functions have been used to provide a robust factorization of the data and a computationally efficient model. There exist a series of tunable parameters in these symmetry functions controlling which specific bond length and bond angle interactions are captured for each symmetry function. For each structure, there are around 70 symmetry functions. Each set of symmetry functions was fed to a neural network with four or five hidden layers each with 64 nodes per layer, to finally predict the energy combination of that atomic species with the total energy of the material. In this ANN, the molecule is also divided into atoms, and summation of the atomic energies gives the total energy of the molecule. In this method, each atom has a specific property, which is unique in the related molecule. For example, the O atom in HNCO and HOCN does not have the same environment. As a result, the atomic energy of the O atom in these molecules is not the same. Hence, the Gaussian descriptors should reproduce the property of the O atom in HNCO and in HOCN. This is because the BPNN architecture relies on calculating fingerprints of the atomic environment using a set of symmetry functions that need to be selected before the fitting procedure can begin.^58^ To determine ideal parameters for fingerprinting, an optimization of the parameters is necessary. Around 35–45 of the total fingerprints correspond to two or three distinct *G*^2^ fingerprints with *r*_s_ = 0, η = {0.0005, 0.005, 0.0015, 0.003,0.01}; *r*_s_ = min *R_ij_*, η = {0.07, 0.015, 0.03, 0.75,1.5}; and , *m* = {0, 1, ···, *n*}, where *n* is the number of intervals in which we have chosen to divide the space,^71^ and . Similarly, two or three different η values need to be optimized and two *G*^3^ and *G*^4^ fingerprint types with ζ = {1, 4}, γ = {−1, 1}, and η parameter {0.01, 0.03, 0.07, 0.20, 0.4}, {6.0613, 12.1227, 18.1840, 24.2454}, and η_s,m_ similar to that used for *G*^2^. With the optimization algorithm used, a search has been carried out in this three-dimensional parameter space, attempting to minimize the prediction error on a test set.^72^ It should be noticed that the PiNN code takes advantage of the evolving TensorFlow^73^ ecosystem, which allows one to access novel optimization methods implemented in its peripheral packages such as K-FAC (Kronecker-factored approximate curvature) for fast training of ANNs.^58^

#### Details Regarding Obtaining the Graph Convolutional Neural Networks (PiNet)

As commented above, the BPNN requires selecting a set of symmetry functions before starting the fitting procedure. On the contrary, in convolutional neural networks, the features are automatically learned via a feature hierarchy. Hierarchical atomic features can be obtained by applying multistage concatenated convolution operations, and this leads to an impressive performance for a variety of systems. Furthermore, the fact that molecular systems can be viewed as fully connected graphs led to the development of graph convolution neural networks (GCNN) able to describe atomic systems.^74^ This is the third ANN variant that we have adapted to explore the tetra atomic isomers of the biogenic [H, C, N, O] system. GCNN is implemented in the PiNN package, and it is known as PiNet. The ingredient of convolution is often recognized as a learnable radial filter that gathers information from neighboring atoms within a cutoff radius *R*_c_ and creates a feature hierarchy. Since the generation of node features includes the element specificity, GCNN has exactly the same subnet for each chemical element. In the structure of PiNet, a series of pairwise interaction (PI) operation and interaction pooling (IP) operation are needed.^58^ Using atomic features such as the nuclear charge Zi as the starting point in the structure of PiNet, the PI are created as a function of the initial atomic features of two atoms and their distance *r_ij_*. The opposite of the PI operation is IP, an operation creating an atomic feature from all of the pairwise interactions associated with that atom. The PI operation is split into three steps: expressing the interatomic distances in a radial basis and activation through the PI layer and the interaction layer. The PI layer is a feed-forward NN generating a weight matrix from the atomic properties, whereas activation through interaction to interaction layer is a feed-forward NN using the information from the previous two steps as input and generating the interaction.^58^

#### Details Regarding Obtaining the Unified Neural Network

In the following, we will use the Sn-SLNN, Sn-BPNN, and Sn-PiNet to denote the separate trained ANNs for tetra atomic isomers of the biogenic [H, C, N, O] system. Here, Sn corresponds to the studied isomers S1–S8. The adjustable parameters of each Sn-SLNN are reported in Table 1, and information about Sn-BPNN and Sn-PiNet including atomic descriptors and hyperparameters are summarized in Tables 2 and 3 for each tetra atomic isomer of the biogenic [H, N, C, O] system.

**Table 1:** Adjustable Parameters and RMSEs of Sn-SLNN^a^

structure	#neurons	#epochs	train	validation	test
S1	80	2000	0.35	0.16	0.18
S2	90	2000	0.69	1.00	1.13
S3	70	500	0.25	0.55	0.62
S4	80	500	0.82	1.07	0.82
S5	80	2000	0.13	0.31	0.19
S6	50	2000	1.13	1.26	1.07
S6*	50	500	0.75	0.40	0.94
S7	90	2000	0.63	0.36	0.41
S8*	100	2000	0.31	1.44	1.63

a The structure names are as in Figure S1; #neurons and #epochs stand for the number of neurons and number of epochs, respectively. Train, validation, and test provide the RMSE value for each set, which are given in kcal/mol. For the S6* and S8* structures, the training range of dihedral angles is 0–180°.

**Table 2:** Hyperparameters for Sn-BPNN^a^

structure	#NN(C,N,O)	#NN(H)	*R*_c_	RMSE
S1	64*4,1	64*4,1	3	0.85
S2	64*5,1	64*4,1	3	0.41
S3	64*4,1	64*4,1	3.5	0.88
S4	64*5,1	64*4,1	3.5	0.56
S5	64*4,1	64*4,1	3.5	0.79
S6	64*5,1	64*4,1	3	0.52
S7	64*4,1	64*4,1	3	0.57
S8	64*4,1	64*4,1	3	0.85

a Structure name is as in Figure S1; #NN(C,N,O) and #NN(H) correspond to the number of atomic neural networks for N, C, and O atoms and the number of atomic neural network for the H atom, respectively, and *R*_c_ corresponds to the optimum cutoff radius for each structure. The chosen sparse_batch value was 2000, whereas max_steps for train and validation set was chosen as 100 000 and 1000, respectively. The RMSE is given in kcal/mol.

**Table 3:** Hyperparameters Defining the Sn-PiNet^a^

S#	*ii*	*pi*	*pp*	*en*	depth	*R*_c_	**#**basis	RMSE
S1	46,36,36,36	46,36,36,36	46,36,36	46,36,36,36,1	2	5	14	0.35
S2	58,58	58,58	58,58	58,58,1	3	7	18	0.35
S3	64,64	64,64	64	64,1	3	5	14	0.22
S4	64,64	64	64,64	64,64,1	6	5.5	10	0.22
S5	64,64	64	64,64	64,64,1	3	4	10	0.32
S6	48	48,48,48,48,48	48	48,1	4	5.5	12	0.53
S7	64,64,64,64,64	64,64	64	64,64,1	5	6.5	11	0.21
S8	38,38	38,38,38	38,38	38,38,1	7	4.5	12	0.35

a S# corresponds to the structure name, whereas *ii*, *pi*, *pp*, and *en* are the number of nodes governing the interaction to interaction NN layer, pairwise interaction NN layer, pairwise pooling NN layer, and atomic NN layer, respectively. Depth corresponds to the number of interaction blocks; *R*_c_ is the cutoff radius, and #basis is the number of basis functions to use. For all networks, we used sparse_batch = 2000 and max_steps for train and validation sets, which were taken as 100 000 and 1000, respectively. RMSE values are in kcal/mol.

To construct a unified neural network (U-ANN) for all of the mentioned eight isomers, one needs to design the unified (U) input data set. To this end, we have first found transition-state structures between each pair of isomers (Figure S2) and we perform relax surface scan calculations with the ORCA V.3.0.3 at the TPSS0/def2-qzvpp level of theory. In this type of calculation, a selected coordinate is kept fixed and the other coordinates are optimized. After relax surface scan calculations, suggested transition states were optimized with the *tsopt* or ‘tsopt’ command to confirm the transition-state criterion that is to exhibit only one imaginary frequency (see Figure S2). Also, the energy of structures is refined at the CCSD(T)/ def2-qzvpp level of theory.

## Results and Discussion

**Figure 3:**
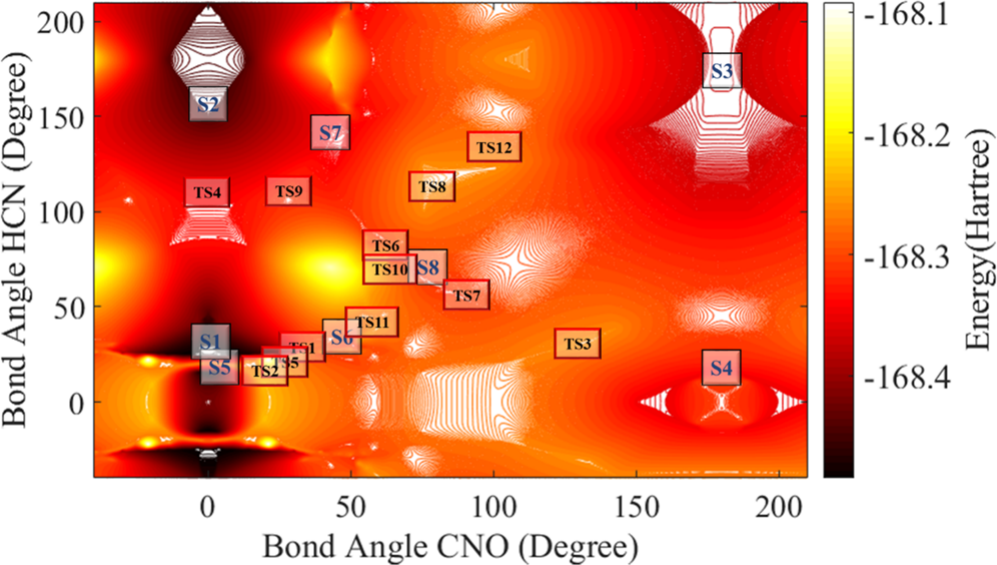
Reference contour of the singlet potential energy surface of S1–S8 structures at the CCSD(T)/def2-qzvpp level of theory. S1–S8 and TS1–TS12 represent the stable structures and transition states, respectively. Energies and bond angles are in atomic unit and degree, respectively. The bond angle variation is between 0 and 180, and the rest of the points were obtained through symmetry.

**Figure 4:**
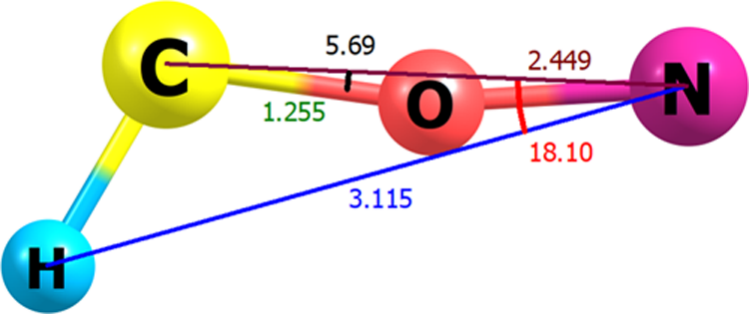
Atomic structure of the HCON isomer as calculated by the tpss/def2-tzvp level of theory.

We start this section by revisiting the available information for the singlet PES for the tetra atomic isomers of the biogenic [H, C, N, O] system. Using traditional methods, previous articles have attempted to provide a potential energy surface describing all members of this family.^34,38,48,52,75^ In this section, we first show that the computational setup described in the previous section accurately reproduces these previous results that are taken as reference to assess the accuracy of the potential energy surfaces here reported based on the use of the artificial neural network method. To this end, after geometry optimization of the eight structures shown in Figure S1 in the SI file, we have searched for transition states governing the isomerization paths between each pair of isomers using the described DFT method and calculated the CCSD(T) energies of the corresponding structures. The obtained results for the eight isomers and twelve transition-state structures are reported in Figure 1. A representation of the PES at the CCSD(T) level of theory is provided in Figure 3, which follows the intelligent presentation of contour plots of this system as reported by Poppinger et al.^38^ The only difference between the present and previous results is in structure S5, which is absent in the previous study.^38^ In addition, we have compared the obtained structures with nine intermediates in the molecular form in the singlet PES as reported by Rey Planells and Ferao^52^ at the B3LYP/def2-TZVP level of theory. Notice, however, that the HCON structure (Figure 4) is not included in the present study because it has a much higher energy than the other ones (167.21 kcal/mol relative to HNCO). Also, we could not find a TS relating any of the other structures to HCON. Apart from this, the contour plot in Figure 3 and the comparison to structures reported by Rey Planells and Ferao^52^ provide a firm basis for the assessment of the constructed ANN-based PES presented in the following subsections.

### Performance of ANN Models of PES for Tetratomic Isomers of the [H, N, C, O] System

To construct the three ANN models described above, the six internal degrees of freedom of the most stable isomer (HNCO) are selected as common coordinates for all tetra atomic isomers of the biogenic [H, C, N, O] system. In a first step, the adjustable parameters of Sn-SLNN have been obtained by looking for a converged performance. Next, the optimal training set has been used to construct the Sn-BPNN and Sn-PiNet networks.

**Figure 5:**
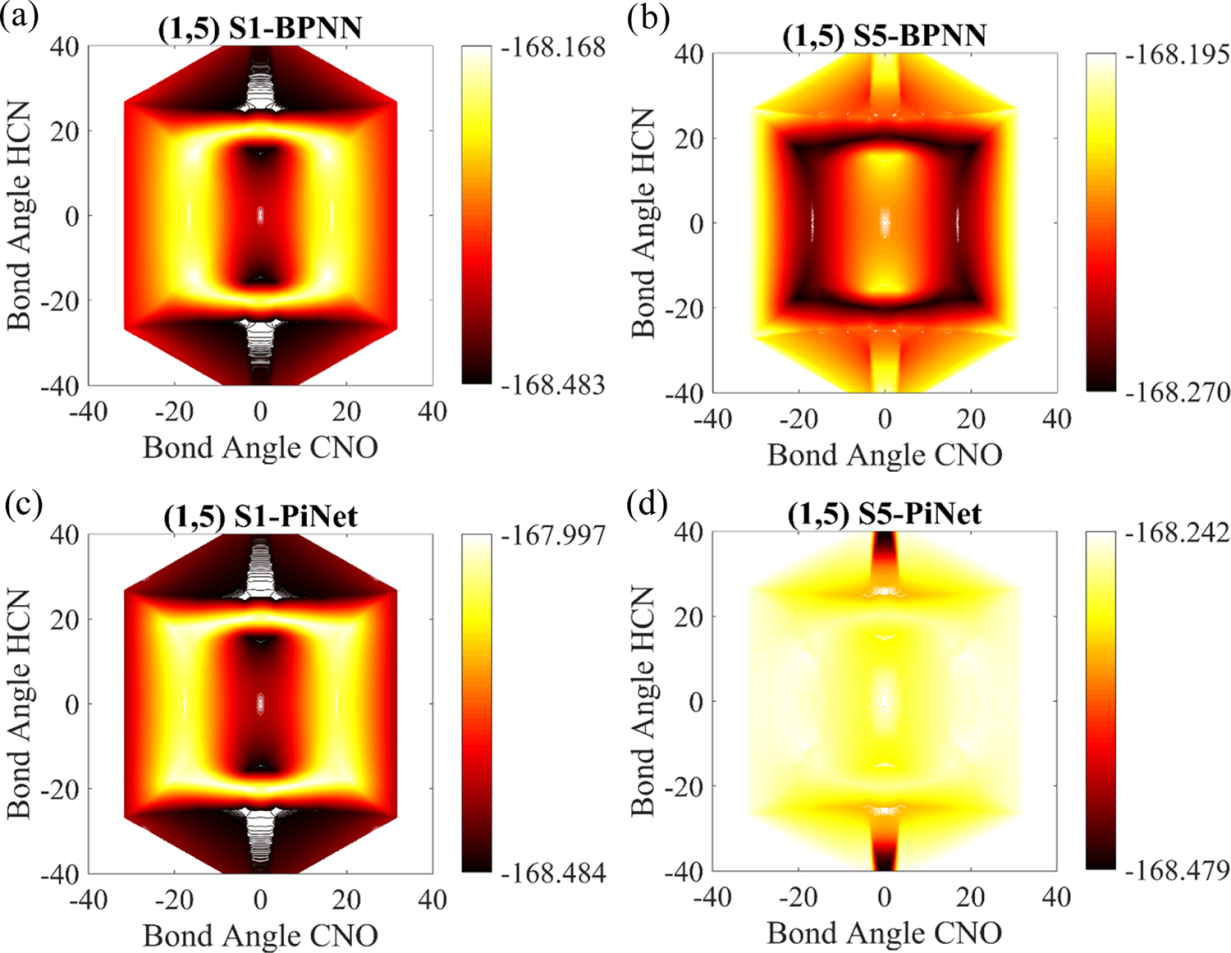
Contour of both S1 and S5 together as reproduced by S1-BPNN (a) and S1-PiNet (c), respectively. Panels b and d correspond to contours of both S1 and S5 together as reproduced by S5 BPNN and S5 PiNet, respectively. The bond angle range covers that of S1 and S5.

In the following, we report eight trained ANNs for structures S1–S8 depicted in Figure S1. The optimized adjustable parameters and the training results of Sn-SLNN, Sn-BPNN, and Sn-PiNet constructed networks are summarized in Tables 1–3, respectively. The performance of each of the three variants of ANNs is established by comparing RMSEs and predicted contour plots for each structure. Contour plots of structures S1–S8 that are reproduced by the constructed Sn-ANNs are depicted in Figures S5–S28 in the SI. From the reference contour plot in Figure 3, it is clear that one of the most complex regions is one where the <HCN and <CNO angles are in the (0, 40) degree range. Since structures S1 and S5 are located in this area, the CCSD(T) contour for this region is zoomed also in Figure S3c in the SI file. The AAN (SLNN, BPNN, and PiNet)-predicted contour plots of individual S1 and S5 and also including both S1 and S5 together are depicted in Figure S4 in the SI file and in Figure 5, respectively.

To assess the performances of the constructed ANNs, we have reproduced the S1 and S5 energies in four cases, namely, reproduction of S1 and S5 energies together by trained S1- and S5-ANNs, respectively, reproduction of S1 and S5 energies together with trained S1-AANs, and reproduction of S1 and S5 energies with trained S5-AANs. The reproduced contour plots are depicted in Figures 5 and S4. The RMSEs of reproduced energies of individual S1 (S5) for S1(S5)-SLNN, S1(S5)-BPNN, and S1(S5)-PiNet are 0.55 kcal/mol (1.26 kcal/mol), 0.62 kcal/mol (0.38 kcal/mol), and 0.33 kcal/mol (0.19 kcal/mol), respectively (Figure S4). Within this strategy, only individual contour plots could be reproduced, which are shown in Figure S4. Reproduced contour plots considering simultaneously S1 and S5 BPNN as well as S1 and S5 PiNet are shown in Figure 5. The reported plots show that the S1 (S5)-SLNN that is trained solely for S1 or for S5 cannot reproduce S1 and S5 contours simultaneously. The RMSEs of the reproduced (S1,S5) data points with just S1 and just S5 BPNN and just S1 and just S5 PiNet are large (22.21 and 124.77 kcal/mol for BPNN and 84.59 and 77.39 kcal/mol for PiNet, respectively). Here, it should be noticed that training data points of each S1(S5) network contain just their own points.

Summing up, reproduced energies using S1(S5)-AANs show that the S1(S5)-SLNN network has a limited ability to reproduce contour plots of S1 and S5 structures all together. The energy range of the training sets for individual S1 and S5 limits the performance of S1 (S5)-SLNN, and also the extrapolation capability of the Sn-SLNN is weak, even if the S1 (S5)-BPNN and S1(S5)-PiNet, which are deep networks, have extended energy ranges for reproducing the energy points due to the considering of physical features in their training process.

Interestingly, the S1 trained network has better performance because S1 is the most stable structure, and the largest energy range is specific to this network, the training set expanding from −168.48456 to −167.74933 au. Contrarily, the energy range of the S5 training set, going from −168.2737 to −166.3290 au, implies that the energy range of S5-AANs does not overlap that of the S1 region. Therefore, the reproduction of energy points of S1 using S5-ANNs is not possible. However, an interesting point is that the PiNet can reproduce a more extended energy range, although with low accuracy. The obtained results in this section give us the promise that PiNet has the potential to provide increased accuracy on increasing the energy range. In the next step, by integrating individual training sets into a unique one and enlarging the energy range, we seek to achieve a unified potential for these isomers.

Based on the discussion above, we have constructed Sn-SLNN, Sn-BPNN, and Sn-PiNet with chemical accuracy for each of the S1–S8 structures with parameters reported in Tables 1–3. We should emphasize that there exist eight common optimal training sets belonging to S1–S8 structures, and they are used to train the three variants of the artificial neural networks.

By examining the reproduced energies for each of the eight studied structures by the three-trained neural networks, it is found that PiNet results excel in quantitative prediction of energies to those predicted by the two other networks. The superior performance of PiNet is due to its particular architecture that has the ability to predict the energy of structures that are not included in the training set. It is also found that expanding the range of the internal coordinate changes in such a way that it includes an extended configuration space improves the performances of the artificial neural networks. Finally, truncation of the repulsion and long-range branch of the individual isomers improves the performances of the ANNs.

### Unified ANN Models of PES of Tetra Atomic Isomers of the [H, N, C, O] System

Taking into consideration the points above, we designed a unified training data set to construct the unified ANN PES (U-SLNN, U-BPNN, U-PiNet) aimed at accurately reproducing the eight tetra atomic isomers of the [H, N, C, O] system and the PES regions around them. To date, we have eight training sets leading to the trained ANNs each providing high quantitative accuracy, especially for the trained structure but, as commented, with not so good predictions for data outside the data-set training region. Now, merging and augmenting these optimal training data sets lead to a unified training data set (U). To further expand the range of internal coordinates, we have added transition-state points in the isomerization pathways to the unified training data set. Therefore, the unified training set contains 2645, 2548, 2617, 2797, 2553, 2809, 3052, and 2326 data points for structures S1–S8, respectively, and 979 1-D points of transition states of isomerization pathways. Furthermore, to eliminate the unfitting data points, it makes sense to constrain the data points of the repulsion and dissociation branches. By imposing the maximum energy threshold of −167.85 au, and restricting the variation of dihedral angles to the molecular plane to values between 0 and 180°, the atomic distances to R1 < 0.8 Å, R2, and R3 < 0.9Å, and removing values for distances larger than 3.5 Å, finally, a total of 17 872 points was selected as a unified training data set (U) containing no unfitting data points.

By means of the U training data set, we first trained SLNN, the simplest of the explored ANN. The input of U-SLNN contains the 2-D to 6-D data points (16263) including all eight structures, which randomly split to 80% for the training set and 20% for the test and validation sets. Moreover, 848 1-D points and 762 points in the transition-state regions have been added to the training set. Unfortunately, even using the unified training data set, the U-SLNN was not able to provide the desired quantitative accuracy of RMSE 92 × 10^–4^ au (5.77 kcal/mol) in predicting the energy of tetra atomic isomers of the biogenic [H, C, N, O] system. Details of the trained U-SLNN are summarized in the first row of Table 4. Therefore, the same procedure was applied to train U-BPNN and U-PiNet; again the unified training data sets were split randomly, 80% for the training set and 20% for the test and validation sets. The best RMSE values for the prediction from U-BPNN and U-PiNet are 24 × 10^–4^ and 9 × 10^–4^ au (1.51, 0.58 kcal/mol), respectively. The optimal hyperparameters for U-BPNN and U-PiNet are reported in Tables 5 and 6, respectively.

**Table 4:** Adjustable Parameters and RMSE of Unified (U) and Improved Unified (IU) SLNN^a^

ANN	#neurons	C#epochs	train RMSE	Val. RMSE	test RMSE
U	220	2000	5.77	7.09	6.02
IU	300	2000	6.84	8.47	8.41

a RMSE values are in kcal/mol.

**Table 5:** Hyperparameters and RMSE of Unified (U) and Improved Unified (IU) BPNN^a^

AAN	#NN(C,N,O)	#NN(H)	*R*_c_	RMSE
U	64*5,1	64*4,1	6	1.51
IU	64*5,1	64*4,1	6	1.47

a #NN(C,N,O) and #NN(H) correspond to the number of atomic neural networks for N, C, and O atoms and the number of atomic neural network for the H atom; *R*_c_ is the cutoff radius. Sparse_batch and max_steps values for train and validation are as in Table 3. RMSE values are in kcal/mol.

**Table 6:** Hyperparameters Defining the Unified (U) and Improved Unified (IU) PiNet, where *ii*, *pi*, *pp*, and *en* Are the Numbers of Nodes Governing the Interaction to Interaction NN Layer, Pairwise Interaction NN Layer, Pairwise Pooling NN Layer, and Atomic NN Layer, Respectively^a^

AAN	*ii*	*pi*	*pp*	*en*	depth	*R*_c_	#basis	RMSE
U	64,64	64*10	64,64	64,64,1	4	5	13	0.58
IU	64,64	64*8	64,64	64,64,1	4	6.6	10	0.49

a Depth corresponds to the number of interaction blocks; *R*_c_ is the cutoff radius, and #basis is the number of basis functions to use. The sparse_batch and max_steps for train and validation set are as in Table 3. RMSE values are in kcal/mol.

To further improve the results by adding data points to the unified training data set, we have examined the training set of separate ANNs, discussed in the previous section. Data inspection shows that except for the dihedral angles’ degree of freedom, all definable physical structures were covered. Consequently, the change in the dihedral angles’ degree of freedom from 0 to 360° was included in the 1-D changes, but for the 2-D to 6-D added, data points were limited to the equilibrium range. The best choice was to add points to change the dihedral angles in higher dimensions. For this purpose, the data structures with 2-D changes for the dihedral angles in the range of 0–360° in the ranges that were far from the equilibrium angle were obtained with a step of 20°. This set of points was fed to the Sn-PiNet as input as this is the ANN with the best qualitative and quantitative performances in reproducing the energies. By adding the new set of points to the unified training data set, an improved unified training data set was obtained, hereafter designated as IU. We have trained the three variants of ANNs using the IU training data set giving rise to IU-SLNN, IU-BPNN, and IU-PiNet. Details of the performance and their details of these new ANNs are reported in Tables 4–6. Interestingly, the IU-PiNet exhibits the best performance not only displaying an RMSE of 7.8 × 10^–4^ au (or 0.49 kcal/mol) but also reproducing the energy for the structures that do not exist in the unified training data set.

**Figure 6:**
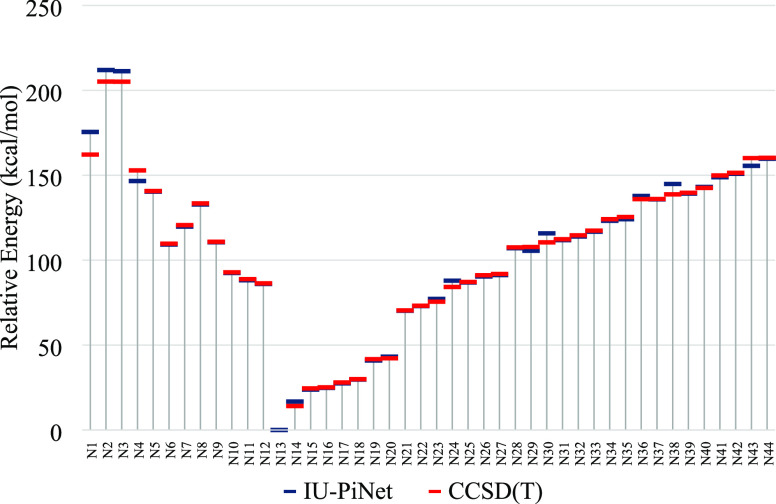
Comparison of the energy values from CCSD(T)/def2-qzvpp calculations and those predicted by the IU-PiNet AAN (kcal/mol) for 12 new structures (N1–N12) from ref (52) and additional structures from the present work.

**Figure 7:**
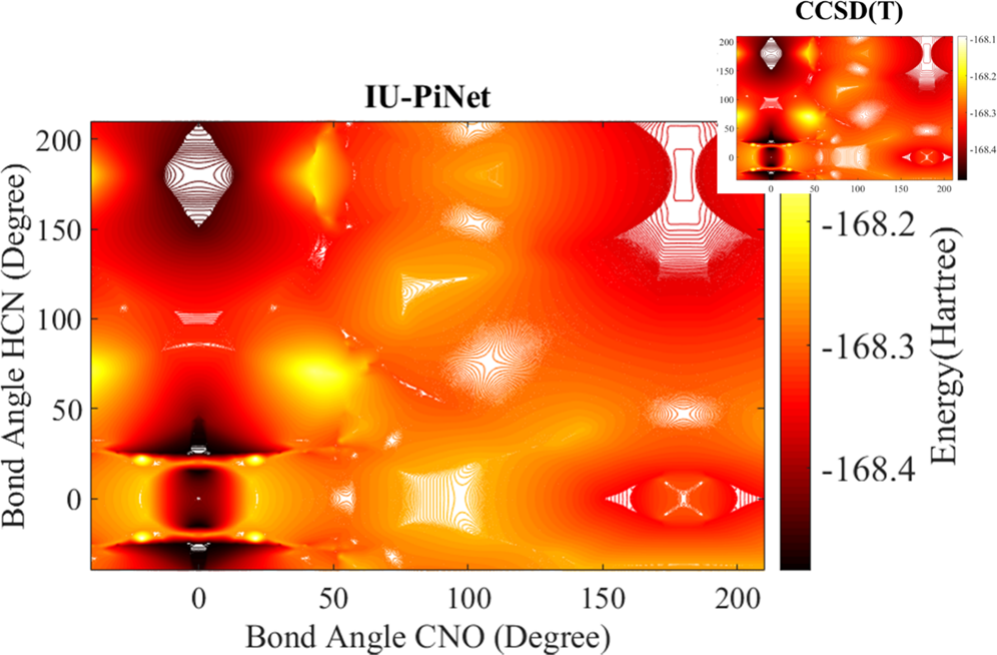
Contour plot as reproduced by IU-PiNet with the one at the CCSD(T)/def2-qzvpp level of theory in the inset for comparison.

To further assess the performance of the IU-PiNet ANN, we compare the reproduced energies with recent results on tetra atomic isomers of the biogenic [H, C, N, O] system.^52^ To this end, 12 new structures from the ref (52) (N1–N12) and 32 new ones (N13–N44) from the present ab initio calculations were selected for a point-by-point comparison. Figure 6 shows that except for the structures N1–N4, the IU-PiNet predicts relative energies of the other eight structures from ref (52) in good agreement with the CCSD(T)/def2-qzvpp reference data. Contrary to N1–N4 structures, the internal coordinates of the structures N5–N12 from ref (52) are in the range of the unified training data set. We recall that ANNs are likely to fail when extrapolating the data outside the trained range. The errors of the IU-PiNet calculated energies relative to the CCSD(T)/def2-qzvpp ones for the structures N1–N4 are 13.4, 6.8, 6.2, and 6.3 kcal/mol, respectively. Inspection of the structural parameters of the structure N1 (Figure 4) exhibiting the largest deviation in reproduced energy shows that both the H–N and N–C distances are greater than 2.4, and according to the mesh grid that was presented in the previous section (Tables S1–S8), these values are outside the range of the unified training data set. As a result, IU-PiNet is less accurate in producing the energy of structure N1. Furthermore, following the work of Poppinger et al.^38^ as mentioned in the previous section, we have designed a new test set that now includes 318 extra data points. Among these 318 data points, more than 90% were not included in the unified training data set. After partial optimization of these new structures, we refined their energy at the CCSD(T)/def2-qzvpp level of theory. Contour plots of the PES of these points are shown in Figure 3. Now, to assess the performances of the IU-PiNet, we reproduced the energy of the later 318 data points. The RMSE for this set is 0.0049 au (3.06 kcal/mol). The reproduced contour is shown in Figure 7. By comparing the contour reported in ref (38) and Figure 7, we have seen that, similar to the CCSD(T) method, the IU-PiNet is able to predict the S5 isomer as a local minimum near the S1 isomer, while it is absent in the corresponding contour in ref (38) because the methods used by these authors do not have the capability to capture this feature of the PES of tetra atomic isomers of the biogenic [H, C, N, O] system. Since the training data set has been designed by the gold standard CCDS(T) method, its abilities have been institutionalized in the trained IU-PiNet. Consequently, the thus-constructed IU-PiNet predicts the S5 isomer with acceptable chemical accuracy. It is worth noting that IU-PiNet performances for the S5 structure and, more importantly, for S1 and S5 structures together are 0.00035 and 0.00107 au (0.22, 0.67 kcal/mol), respectively, while for S1 and S5 structures, the performance of the ANN trained just for S1 is 0.1348 au, as commented in the previous section. Finally, in Figure 6, from point N13 onward, we have compared the relative stability energy of the randomly selected points from the later test set that includes the mentioned new 318 data. The obtained RMSE for the later test set is 0.0049 au (3.06 kcal/mol). Note that some points in Figure 6 have a deviation in the range of 1–7 kcal/mol. These points correspond to the structures where their internal coordinates are out of the range of the unified training data set.

## Conclusions

The present work reports the direct fit of CCSD(T)/def2-qzvpp ab initio data into a six-dimensional (6-D) PES using the SLNN, BPNN, and PiNet artificial neural networks. We have improved the performances of the artificial neural networks using computational chemistry-based preprocessing and chemical intuition. The quality and volume of the training data are the same for the three ANN variants. Even with this identical starting set of data, the ANN architecture and tuning of hyperparameters affect the accuracy of the obtained PES, which makes it difficult to compare the quality and performance of the various potentials that are derived. The present work provides evidence that some differences in performances are related to the ANN model, but the performance of ANN is also related to the atomic environment descriptor and the machine learning methodology, which is important in complex systems. For example, SLNN converges to substantially high accuracies for individual isomers, but it has a poor performance in the unified model.

In the SLNN, the fitting procedure is done with exponential neurons and the quality of the fit depends on the cutoff energy and the number of neurons. Due to the presence of the validation set to prevent overfitting, the optimization of the fit RMSE eventually stops improving with an increased number of neurons. Also, the quantitative performances of BPNN are similar to those of SLNN. In addition, unified BPNN has a better performance than U-SLNN. The third variant, PiNet, exhibits good qualitative and quantitative performance and one additional advantage that, even if it has been trained for individual isomers, it qualitatively predicts the energy of other studied isomers. This is because PiNet takes into consideration the local environment properties of atoms through the NN architecture, and these features are extracted from data via an iterative procedure. Consequently, the unified graph convolution-based IU-PiNet NN shows improved performances when compared to IU-SLNN and IU-BPPN (see Table 7). The quantitative accuracy achieved by IU-PiNet is 7.8 × 10^–4^ au (0.49 kcal/mol), significantly better than those of IU-SLNN and IU-BPNN. The reliability of PiNet for isomer energy predictions is demonstrated by the contour plot in Figure 7.

**Table 7:** RMSE for the U and IU ANNs Trained from SLNN, BPNN, and PiNet^a^

	SLNN	BPNN	PiNet
U	6.02	1.51	0.58
IU	8.41	1.47	0.49

a Values are kcal/mol.

Furthermore, it turns out that the unified PiNet-derived PES model behaves very similarly to the separate models. Although for each trained NN, many different parametrizations are obtained, they are very similar in terms of RMSE. A comparison of RMSEs for the separate and unified ANN models (Figure S29) shows that, in all cases, the PiNet is more efficient than SLNN and BPNN, especially in unified models. Summing up, we presented a new unified six-dimensional potential energy surface for tetra atomic isomers of the biogenic [H, N, C, O] system able to describe a limited region, including the equilibrium geometry of all isomers as well as the transition states for unimolecular isomerizations. Despite the added complexity, our model achieves a mean absolute error of 0.315 kcal mol^–1^. The present work places the PiNet model as a versatile framework for understanding complex quantum mechanical systems based on high-throughput electronic structure calculations that can be extended to derive other PES of the [H, N, C, O] system, particularly, to model the first triplet state.

## Supporting Information Available

Additional information regarding the regular mesh grids (RMGs); Tables S1–S8, RMG information for structures S1–S8; Tables S9–S16, symmetry function details of Sn-BPNN; Figure S1, eight optimized structures at local minimum of the singlet potential energy surface of the biogenic [H, C, N, O] system; Figure S2, transition-state structures in singlet PES are related to isomerization paths; Figure S3, contours of S1 and S5 individually as well as together at the CCSD(T)/def2-qzvpp level of computation; Figure S4, reproduced contour of S1 and S5 by S1(S5)-SLNN, BPNN, PiNet; Figures S5–S28, contour plots for the different isomers as reproduced by the different individual AAN models; Figure S29, comparison of the RMSEs for Sn-SLNN, Sn-BPNN, Sn-PiNet, U(IU)-SLNN, U(IU)-BPNN, and U(IU)-PiNet networks; all CCSD(T) energies and internal coordination of the data sets are available in https://github.com/Nazari-Fariba (PDF)
