# Supplementary material for: Artificial Neural Network-Derived Unified Six-Dimensional Potential Energy Surface for Tetra Atomic Isomers of the Biogenic [H, C, N, O] System
Source: J Chem Theory Comput. 2023 Feb 3;19(4):1186–96. doi: 10.1021/acs.jctc.2c00915 (PMC9979606; doi:10.1021/acs.jctc.2c00915)
Supplement: Supplementary file 1 — ct2c00915_si_001.pdf [file ct2c00915_si_001.pdf]

Artificial Neural Network Derived Unified Six-Dimensional Potential Energy Surface for Tetra Atomic Isomers of the biogenic [H, C, N, O] System

Fatemeh Arab,[1] Fariba Nazari,[1,2] Francesc Illas[3]

[1]*Department of Chemistry, Institute for Advanced Studies in Basic Sciences, Zanjan 45137-66731, Iran*

[2]*Center of Climate Change and Global Warming, Institute for Advanced Studies in Basic Sciences, Zanjan 45137-66731, Iran*

[3]*Departament de Ciència de Materials i Química Física & Institut de Química Teòrica i Computacional (IQTCUB), Universitat de Barcelona,C/Martí i Franquès 1, 08028 Barcelona, Spain*

Table of Content

1. Additional information regarding the regular mesh grids (RMGs). Tables S1 to S8 report the RMGs information for structures S1 to S8

2. Symmetry function (SF) details of Sn-BPNN: Tables S9-Table S16

3. Additional figures

Figure S1. Eight optimized structures at local minimum of singlet potential energy surface of the biogenic [H, C, N, O] system.

Figure S2. Transition state structures in singlet PES are related to isomerization paths.

Figure S3. Contour of S1, S5 and S1, S5 together at CCSD(T)/def2-qzvpp level of computation. The bond angles range covers that of individual S1 and S5. a, b and c are contour of individual S1, S5 and S1, S5 together, respectively.

Figure S4. Reproduced contour of S1(a, c, e) and S5 (b, d, f) by S1(S5)-SLNN, BPNN, PiNet, respectively. Figure S5-S28 contour plots for the different isomers as reproduced by the different individual AAN models.

Figure S29. Comparison of the RMSEs for Sn-SLNN, Sn-BPNN, Sn-PiNet, U(IU)-SLNN, U(IU)-BPNN and U(IU)-PiNet networks.

Table S1. RMGs information for S1

	Eq.	min-1D	max-1D	min-2D	max-2D	min-3D	max-3D	min-4D	max-4D	min-5d,6D	max-5D,6D
S1	1.002	0.602	4.002	0.702	1.352	0.802	1.202	0.852	1.152	0.852	1.152
	1.207	0.807	4.007	0.907	1.582	1.007	1.407	1.057	1.357	1.057	1.357
	1.158	0.858	4.058	0.858	1.533	0.958	1.358	1.008	1.308	1.008	1.308
	124.696	34.696	174.696	104.696	144.696	104.696	144.696	117.196	132.196	117.196	132.196
	172.697	52.697	172.697	132.697	177.697	132.697	172.697	140.23	180.197	165.23	180.197
	180	0	360	140	220	160	200	165	195	165	195

Table S2. RMGs information for S2

	Eq.	min-1D	max-1D	min-2D	max-2D	min-3D	max-3D	min-4D,5D	max-4D,5D	min-6D	max-6D
S2	2.945	2.345	3.445	2.345	3.295	2.445	3.145	2.795	3.095	2.795	3.095
	1.149	0.849	1.499	0.849	1.499	0.949	1.349	0.999	1.299	0.999	1.299
	1.291	0.991	2.291	0.991	1.641	1.091	1.491	1.141	1.441	1.141	1.441
	15.741	10.741	30.741	10.741	30.741	10.741	25.741	8.241	23.241	8.241	23.241
	176.508	136.508	176.508	136.508	176.508	136.508	176.508	159.008	176.508	159.008	176.508
	179.871	9.871	359.871	139.871	199.871	159.871	199.871	164.871	194.871	164.871	194.871

Table S3. RMGs information for S3

	Eq.	min-1D	max-1D	min-2D	max-2D	min-3D	max-3D	min-4D,5D	max-4D,5D	min-6D	max-6D
S3	2.207	1.807	4.307	1.907	2.557	2.007	2.407	2.057	2.357	2.057	2.357
	1.149	0.749	1.549	0.849	1.499	0.949	1.349	0.999	1.299	0.999	1.299
	2.342	1.942	4.442	2.042	2.692	2.142	2.542	2.192	2.492	2.192	2.492
	1.079	1.079	158.579	1.079	66.079	1.079	41.921	1.079	18.421	8.579	18.421
	1.017	1.017	158.517	1.017	66.017	1.017	41.983	1.017	18.483	8.517	18.483
	179.999	9.999	359.999	159.999	194.999	159.999	199.999	164.999	194.999	164.999	194.999

Table S4. RMGs information for S4

	Eq.	min-1D	max-1D	min-2D	max-2D	min-3D	max-3D	min-4D,5D	max-4D,5D	min-6D	max-6D
S4	1.853	1.453	2.653	1.45	2.203	1.453	2.053	1.703	2.003	1.703	2.003
	1.182	0.882	1.482	0.882	1.532	0.982	1.382	1.032	1.332	1.032	1.332
	2.514	0.126	3.014	2.114	2.864	2.314	2.714	2.364	2.664	2.364	2.664
	158.968	133.968	173.968	128.968	173.968	138.968	168.968	151.468	166.468	151.468	166.468
	5.126	0.126	27.626	0.126	20.126	2.626	15.126	5.126	22.626	12.626	22.626
	179.962	19.962	339.962	139.962	199.962	159.962	199.962	164.962	194.962	164.962	194.962

Table S5. RMGs information for S5

	Eq.	min-1D	max-1D	min-2D	max-2D	min-3D	max-3D	min-4D,5D,6D	max-4D,5D,6D
S5	1.022	0.622	3.122	0.722	1.372	0.822	1.222	0.872	1.172
	2.555	2.155	4.655	2.255	2.905	2.355	2.755	2.405	2.705
	1.151	0.751	1.951	0.851	1.501	0.951	1.351	1.001	1.301
	104.512	64.512	179.512	84.512	124.512	84.512	124.512	97.012	112.012
	8.346	3.346	38.346	3.346	23.346	1.654	28.346	0.846	15.846
	0	0	360	0	350	0	350	0	345

Table S6. RMGs information for S6

	Eq.	min-1D	max-1D	min-2D	max-2D	min-3D	max-3D	min-4D,5D,6D	max-4D,5D,6D
S6	1.021	0.621	3.121	0.721	1.371	0.821	1.221	0.871	1.171
	1.437	1.037	3.537	1.137	1.787	1.237	1.637	1.287	1.587
	1.27	0.87	3.37	0.97	1.62	1.07	1.47	1.12	1.42
	106.879	66.879	174.379	86.879	131.879	86.879	126.879	99.379	114.379
	68.047	28.047	105.547	43.047	93.047	48.047	88.047	60.547	75.547
	94.139	4.139	354.139	54.139	114.139	74.139	114.139	79.139	109.139

Table S7. RMGs information for S7

	Eq.	min-1D	max-1D	min-2D	max-2D	min-3D	max-3D	min-4D,5D,6D	max-4D,5D,6D
S7	2.196	1.796	4.296	1.896	2.546	1.996	2.396	2.046	2.346
	1.243	0.843	3.343	0.943	1.593	1.043	1.443	1.093	1.393
	1.301	0.901	3.401	1.001	1.651	1.101	1.501	1.151	1.451
	17.647	2.647	175.147	7.647	32.647	7.647	37.647	10.147	25.147
	85.366	45.366	175.366	70.366	100.366	65.366	105.366	77.866	92.866
	179.998	9.998	359.998	119.998	269.998	159.998	199.998	164.998	194.998

Table S8. RMGs information for S8

	Eq.	min-1D	max-1D	min-2D	max-2D	min-3D	max-3D	min-4D,5D,6D	max-4D,5D,6D
S8	2.005	1.305	3.005	1.505	2.355	1.605	2.205	1.855	2.155
	1.233	0.833	2.033	0.933	1.583	1.033	1.433	1.083	1.383
	1.687	1.287	2.287	1.387	2.037	1.487	1.887	1.537	1.837
	78.932	43.932	88.932	58.932	88.932	58.932	98.932	71.432	86.432
	62.062	47.062	69.562	47.062	69.562	42.062	67.062	54.562	69.562
	28.656	13.656	33.656	18.656	48.656	18.656	48.656	13.656	43.656

Table S9. Symmetry function details of S1-BPNN.

	SF Type	Zi of atoms	Other parameters	ζ	η
S1	G2	1,7<br>6,7<br>6,8<br>7,8<br>6,1	Rs=0.0		0.0005,0.005,0.0015,0.003,0.01
	G2	1,7<br>6,7<br>6,8<br>7,8<br>6,1	Rs=0.6, 0.7, 0.8, 1.4, 1.8		0.07,0.015,0.03,0.75,1.5
	G3,G4	ALL	lambd = -1.0,1.0	1.0,4.0	0.01,0.03,0.07,0.20,0.4
	G3,G4	ALL	lambd = -1.0,1.0	1.0,4.0	6.06,12.12,18.18,24.24

Table S10. Symmetry function details of S2-BPNN.

	SF Type	Zi of atoms	Other parameters	ζ	η
S2	G2	1,8<br>6,8<br>6,7<br>1,6<br>7,8	Rs= 0.0		0.0005,0.005,0.0015,0.003,0.01
	G2	1,7<br>6,7<br>6,8<br>7,8<br>6,1	Rs= 0.6, 0.7, 0.8, 1.3, 1.9		0.07,0.015,0.03,0.75,1.5
	G2	ALL	Rs= 3.0,2.7,2.4,2.1,1.9,1.7		9.67,12.13,15.22,19.09,23.95,30.05
	G3,G4	ALL	lambd = -1.0,1.0	1.0,4.0	0.11,0.15,0.21,0.29,0.40

Table S11. Symmetry function details of S3-BPNN.

	SF Type	Zi of atoms	Other parameters	ζ	η
S3	G2	1,6<br>6,7<br>8,7<br>1,7<br>6,8	Rs= 0.0		0.0005,0.005,0.0015,0.003,0.01
	G2	1,6<br>6,7<br>8,7<br>1,7	Rs= 0.6, 0.7, 0.7, 1.7		0.07,0.015,0.03,0.75,1.5
	G2	ALL	Rs= 3.5,3.2,2.8,2.6,2.3,2.1		8.7,10.7,13.1,16.1,19.7,24.2
	G3,G4	ALL	lambd = -1.0,1.0	1.0,4.0	0.08,0.11,0.15,0.21,0.29

Table S12. Symmetry function details of S4-BPNN.

	SF Type	Zi of atoms	Other parameters	ζ	η
S4	G2	1,8<br>8,7<br>8,7<br>1,7<br>6,8	Rs=0.0		0.0005,0.005,0.0015,0.003,0.01
	G2	1,8<br>8,7<br>8,7<br>1,7<br>6,8	Rs=0.6,0.9,0.7, 1.3, 1.9		0.07,0.015,0.03,0.75,1.5
	G2	ALL	Rs= 3.5,3.2,2.8,2.6,2.3,2.1		8.7,10.7,13.1,16.1,19.7,24.2
	G3,G4	ALL	lambd = -1.0,1.0	1.0,4.0	0.08,0.11,0.15,0.21,0.29

Table S13. Symmetry function details of S5-BPNN.

	SF Type	Zi of atoms	Other parameters	ζ	η
S5	G2	1,7<br>8,7<br>8,6<br>1,8<br>6,8	Rs= 0.0		0.0005,0.005,0.0015,0.003,0.01
	G2	1,7<br>8,7<br>8,6<br>1,8<br>6,8	Rs= 0.6,0.9,0.8, 1.3, 2.0		0.07,0.015,0.03,0.75,1.5
	G2	ALL	Rs= 3.5,3.2,2.8,2.6,2.3,2.1		8.7,10.7,13.1,16.1,19.7,24.2
	G3	ALL	lambd = -1.0,1.0	1.0,4.0	6.06,12.12,18.18,24.24
	G3,G4	ALL	lambd = -1.0,1.0	1.0,4.0	0.08,0.11,0.15,0.21,0.29
	G4		lambd = -1.0,1.0	1.0	6.06,12.12,18.18,24.24

Table S14. Symmetry function details of S6-BPNN.

	SF Type	Zi of atoms	Other parameters	ζ	η
S6	G2	1,7<br>8,7<br>8,6<br>1,8<br>6,8	Rs=0.0		0.0005,0.005,0.0015,0.003,0.01
	G2	1,7<br>6,7<br>8,7<br>1,6<br>7,8	Rs=0.6,0.9,0.8, 1.4, 1.0		0.07,0.015,0.03,0.75,1.5
	G2	ALL	Rs= 3.0,2.7,2.4,2.1,1.9,1.7		9.67,12.13,15.22,19.09,23.95,30.05
	G3,G4	ALL	lambd = -1.0,1.0	1.0,4.0	0.11,0.15,0.21,0.29,0.40

Table S15. Symmetry function details of S7-BPNN.

	SF Type	Zi of atoms	Other parameters	ζ	η
S7	G2	1,6<br>6,8<br>6,7<br>7,8<br>8,1	Rs= 0.0		0.0005,0.005,0.0015,0.003,0.01
	G2	1,6<br>6,8<br>6,7<br>7,8<br>8,1	Rs= 0.6, 0.7, 0.8, 1.5, 1.5		0.07,0.015,0.03,0.75,1.5
	G3,G4	ALL	lambd = -1.0,1.0	1.0,4.0	0.01,0.03,0.07,0.20,0.4
	G3,G4	ALL	lambd = -1.0,1.0	1.0,4.0	6.06,12.12,18.18,24.24

Table S16. Symmetry function details of S8-BPNN.

	SF Type	Zi of atoms	Other parameters	ζ	η
S8	G2	1,8<br>8,7<br>7,6<br>1,7<br>6,8	Rs= 0.0		0.0005,0.005,0.0015,0.003,0.01
	G2	1,8<br>8,7<br>7,6<br>1,7<br>6,8	Rs= 0.6,1.0,0.8, 1.1, 1.5		0.07,0.015,0.03,0.75,1.5
	G2	ALL	Rs= 3.0,2.7,2.4,2.1,1.9,1.7		9.67,12.13,15.22,19.09,23.95,30.05
	G3,G4	ALL	lambd = -1.0,1.0	1.0,4.0	6.06,12.12,18.18,24.24
	G3,G4	ALL	lambd = -1.0,1.0	1.0,4.0	0.11,0.15,0.21,0.29,0.40

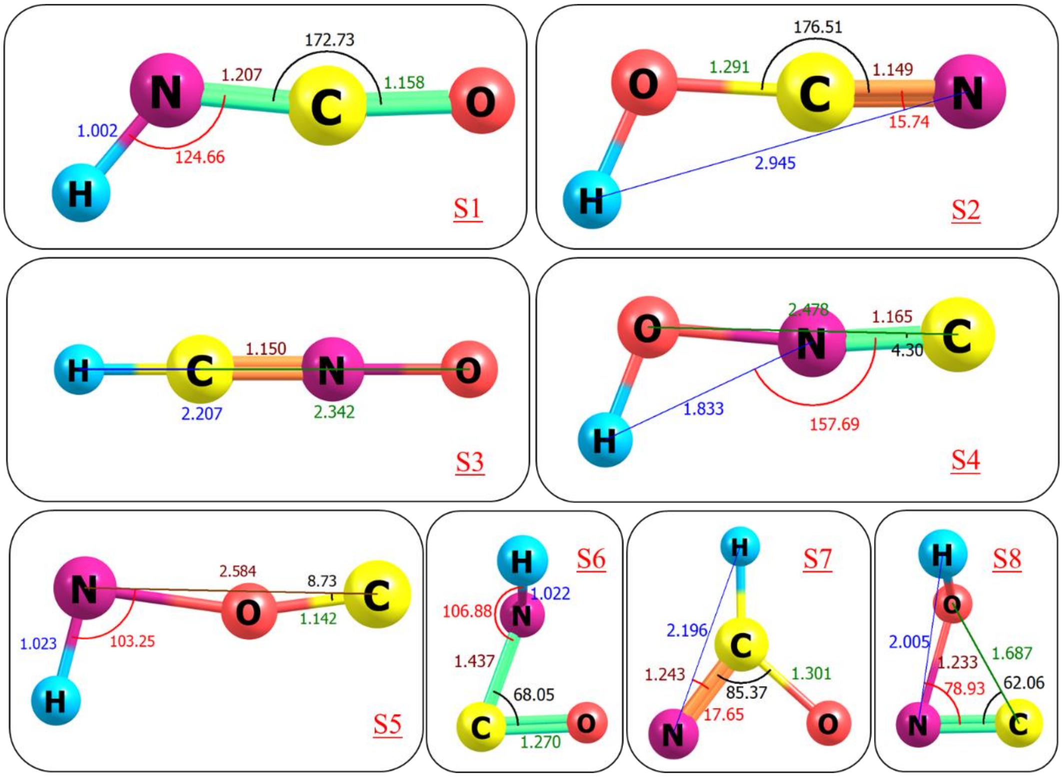

Figure S1. Eight optimized structures at local minimum of singlet potential energy surface of the biogenic [H, C, N, O] system.

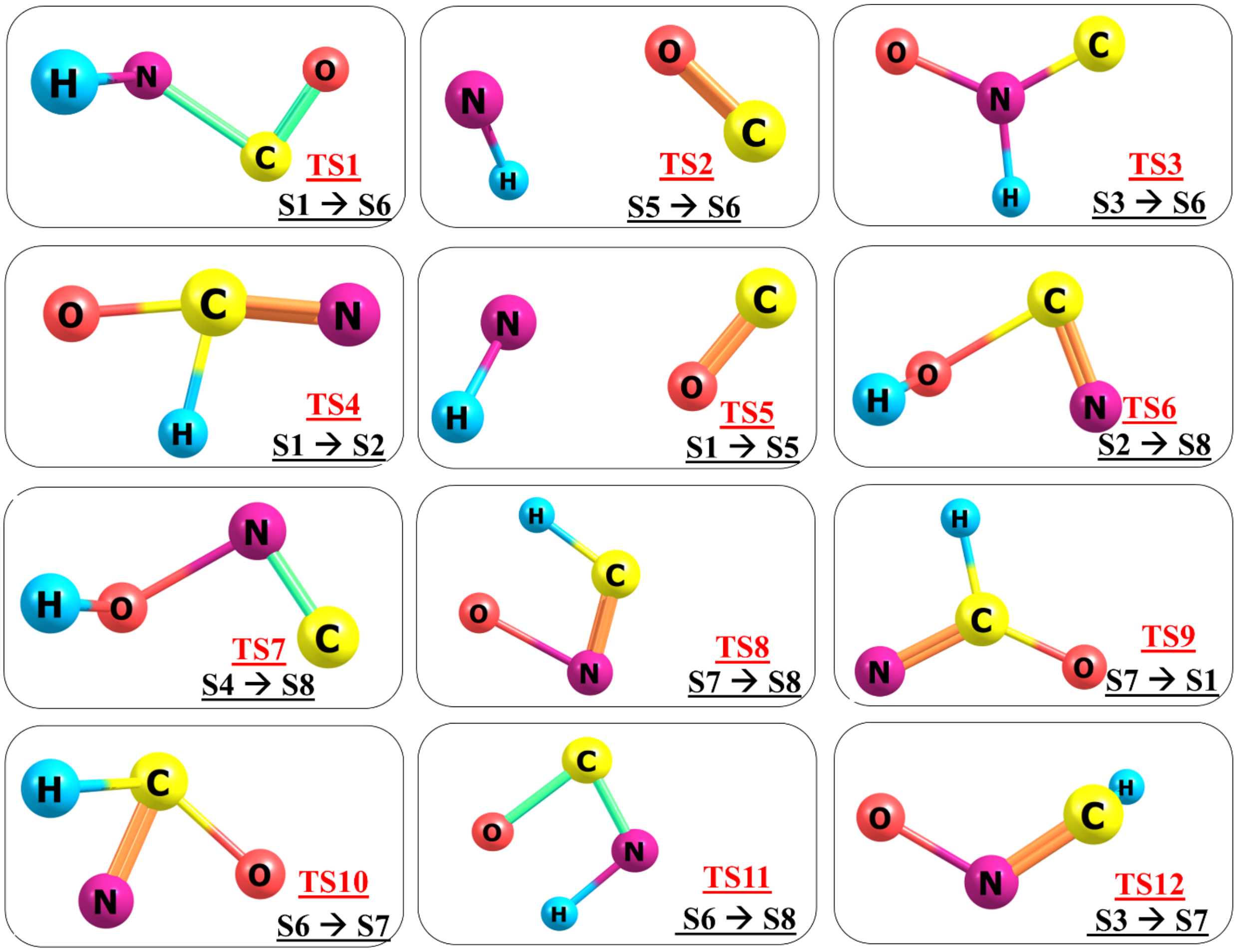

Figure S2. Transition state structures in singlet PES, which are related to isomerization path.

a) b) c)

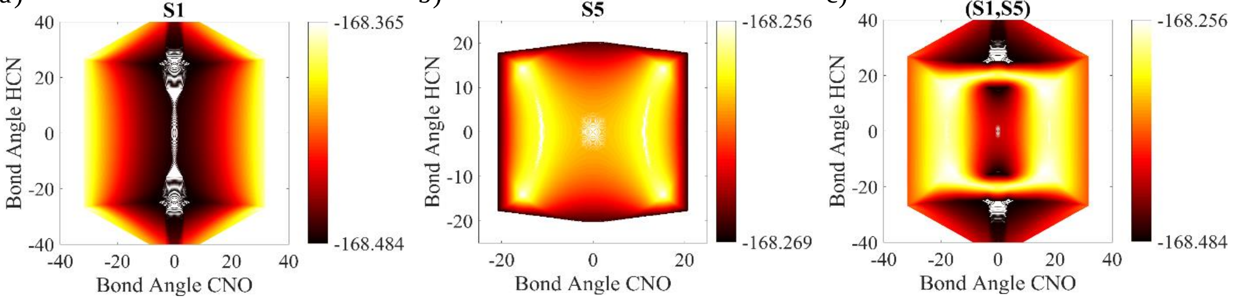

Figure S3. Contour of S1, S5 and S1, S5 together at CCSD(T)/def2-qzvpp level of computation. The bond angles range covers that of individual S1 and S5. a, b and c are contour of individual S1, S5 and S1, S5 together.

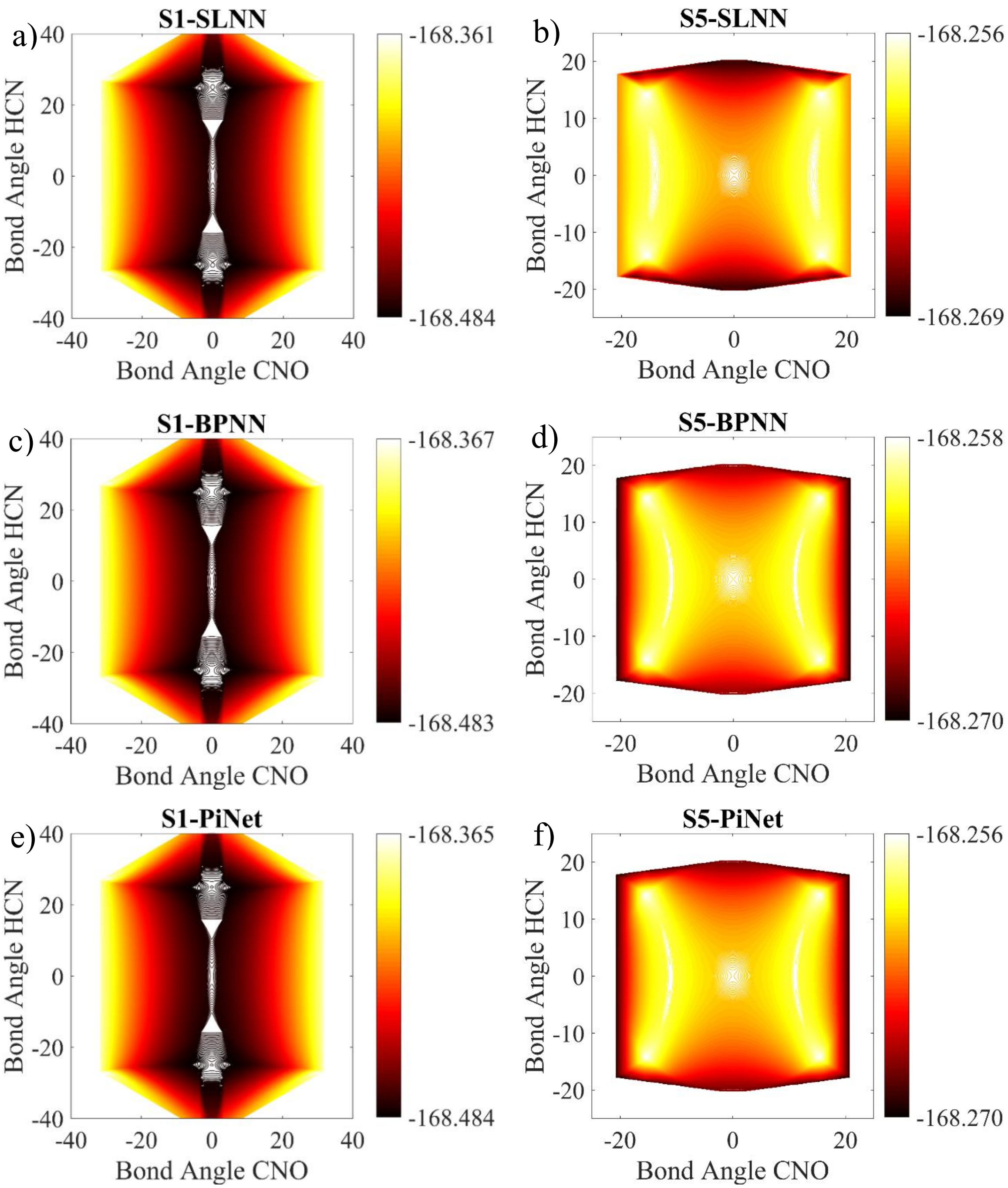

Figure S4. Reproduced contour of S1(a, c, e) and S5 (b, d, f) by S1(S5)-SLNN, BPNN, PiNet, respectively.

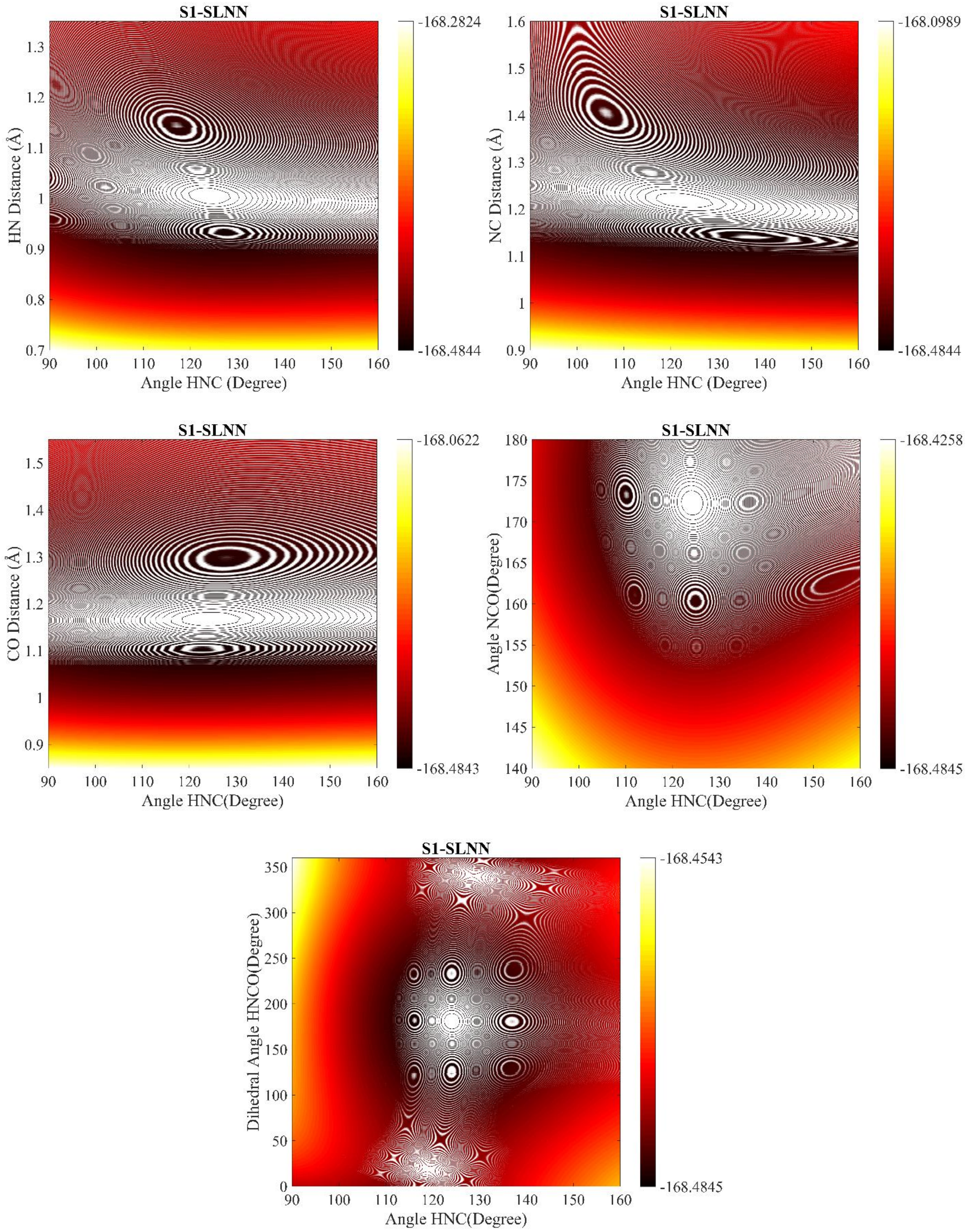

Figure S5. S1-SLNN reproduced contours. Energy unite is Hartree.

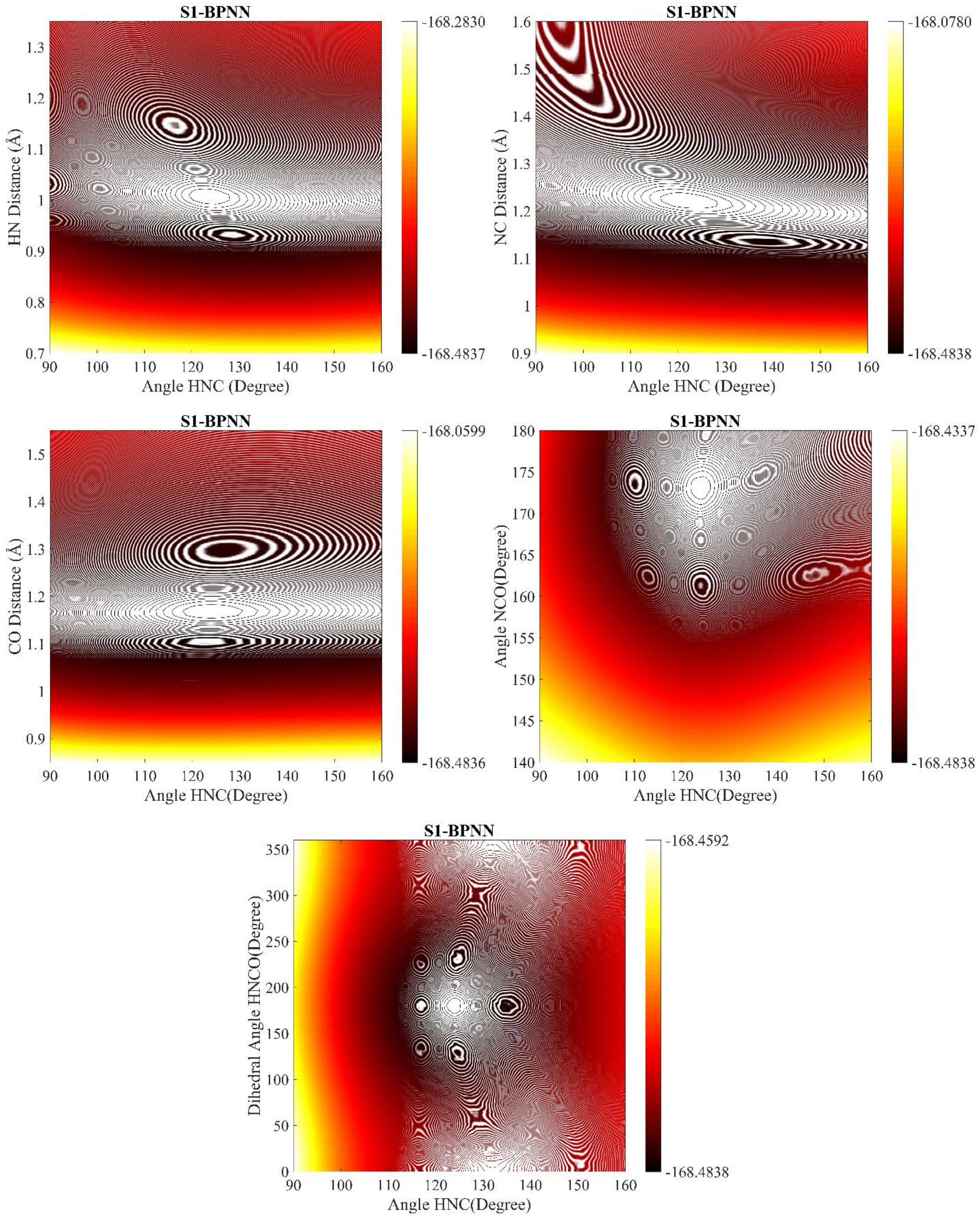

Figure S6. S1-BPNN reproduced contours. Energy unite is Hartree.

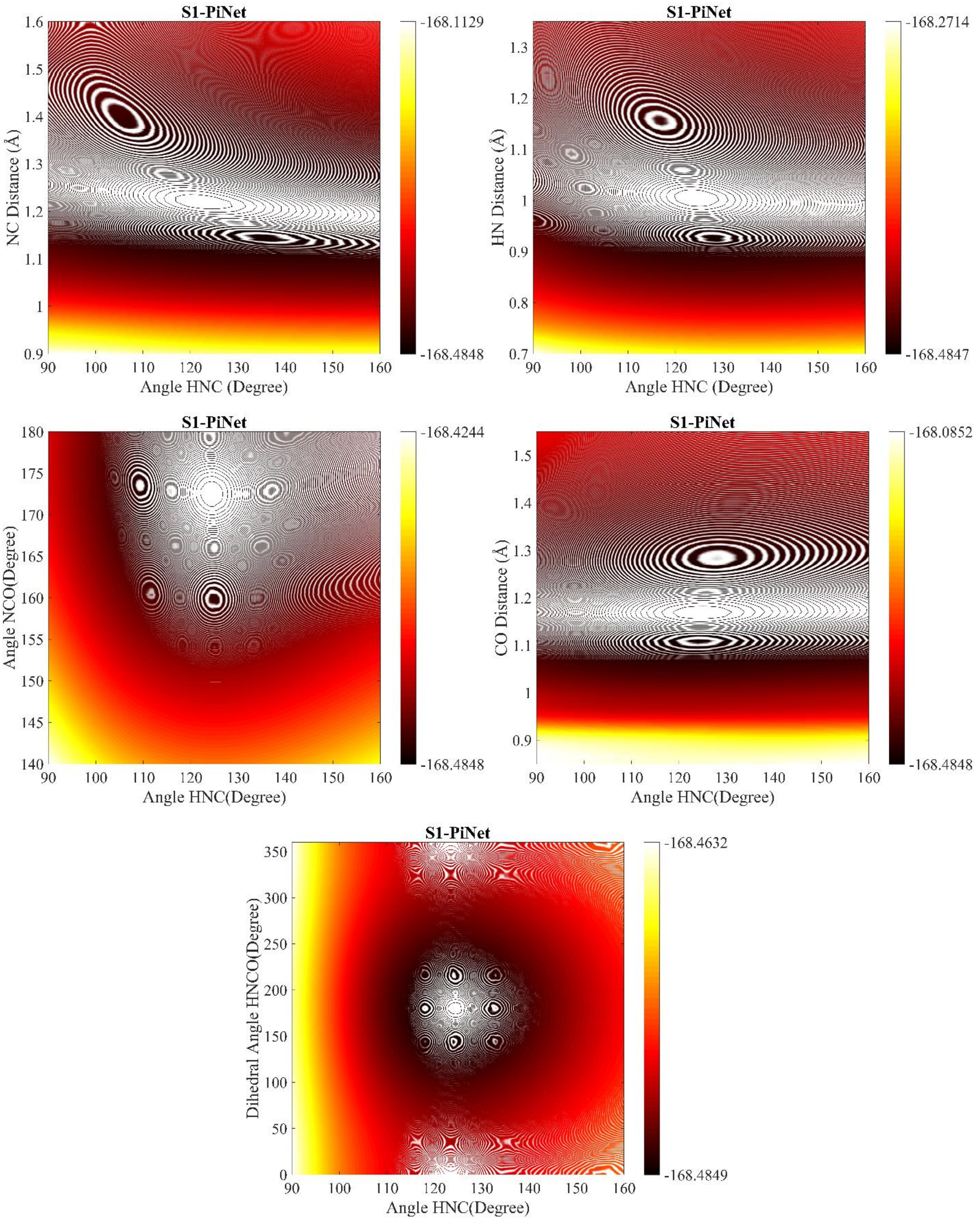

Figure S7. S1-PiNet reproduced contours. Energy unite is Hartree.

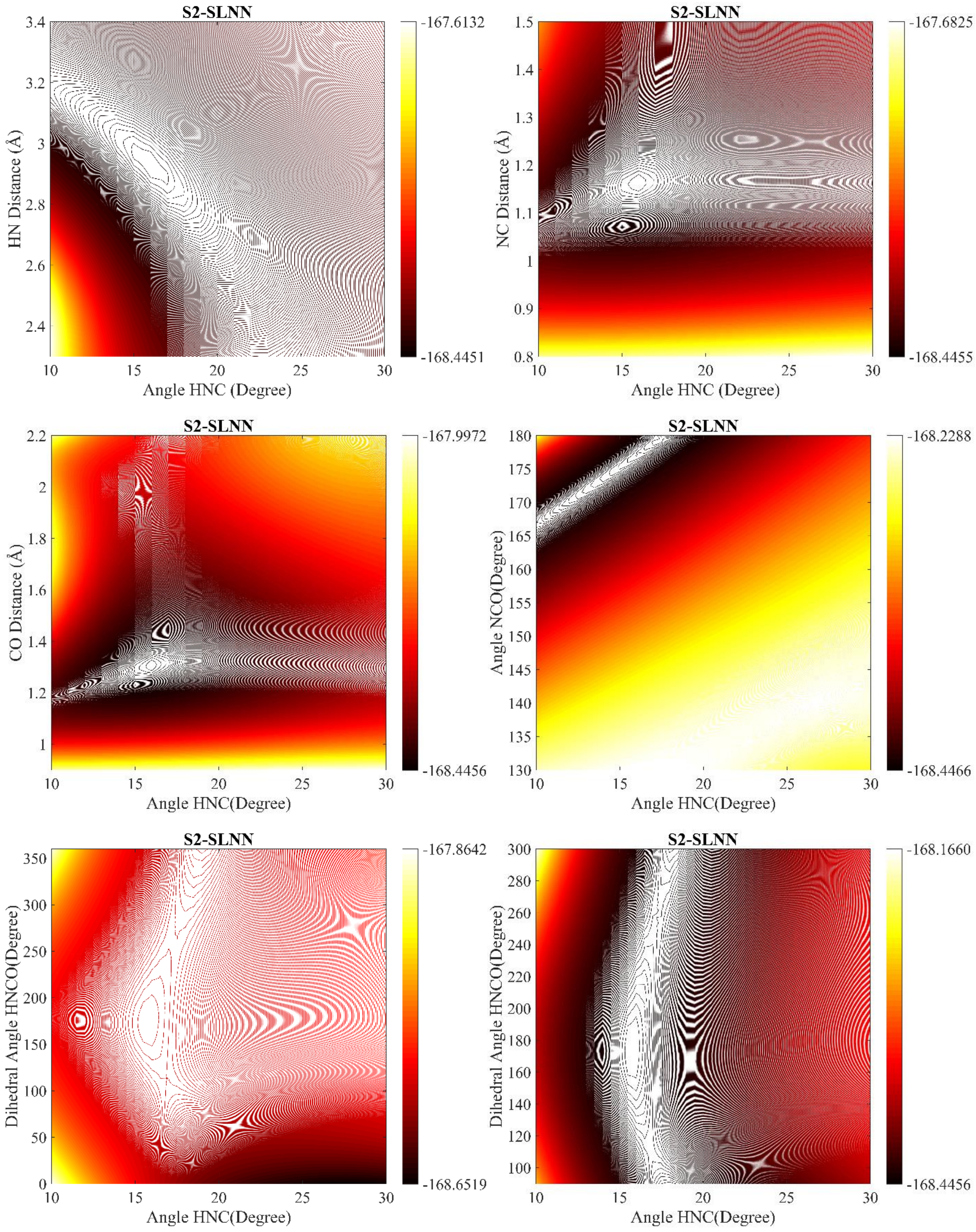

Figure S8. S2-SLNN reproduced contours. Energy unite is Hartree.

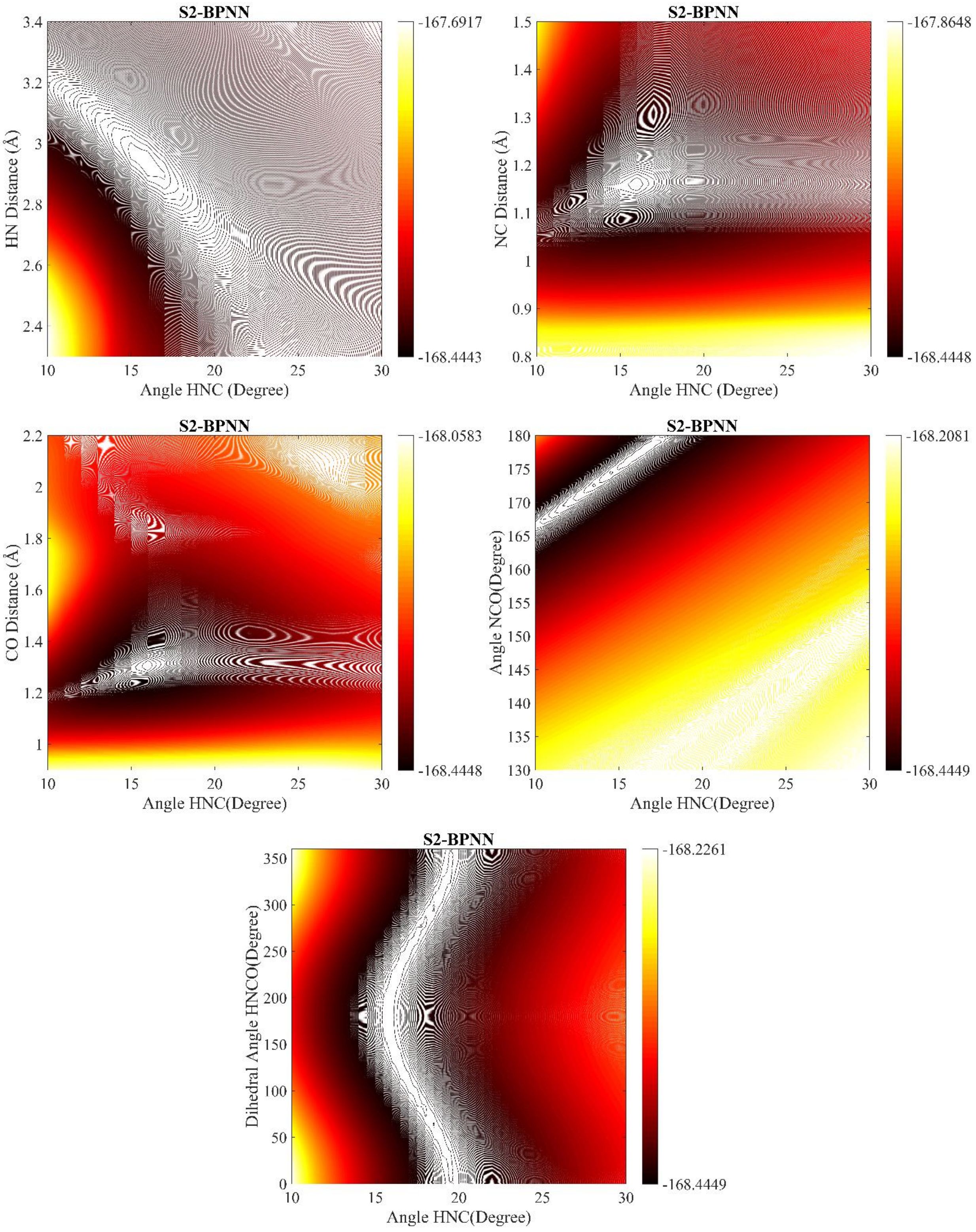

Figure S9. S2-BPNN reproduced contours. Energy unite is Hartree.

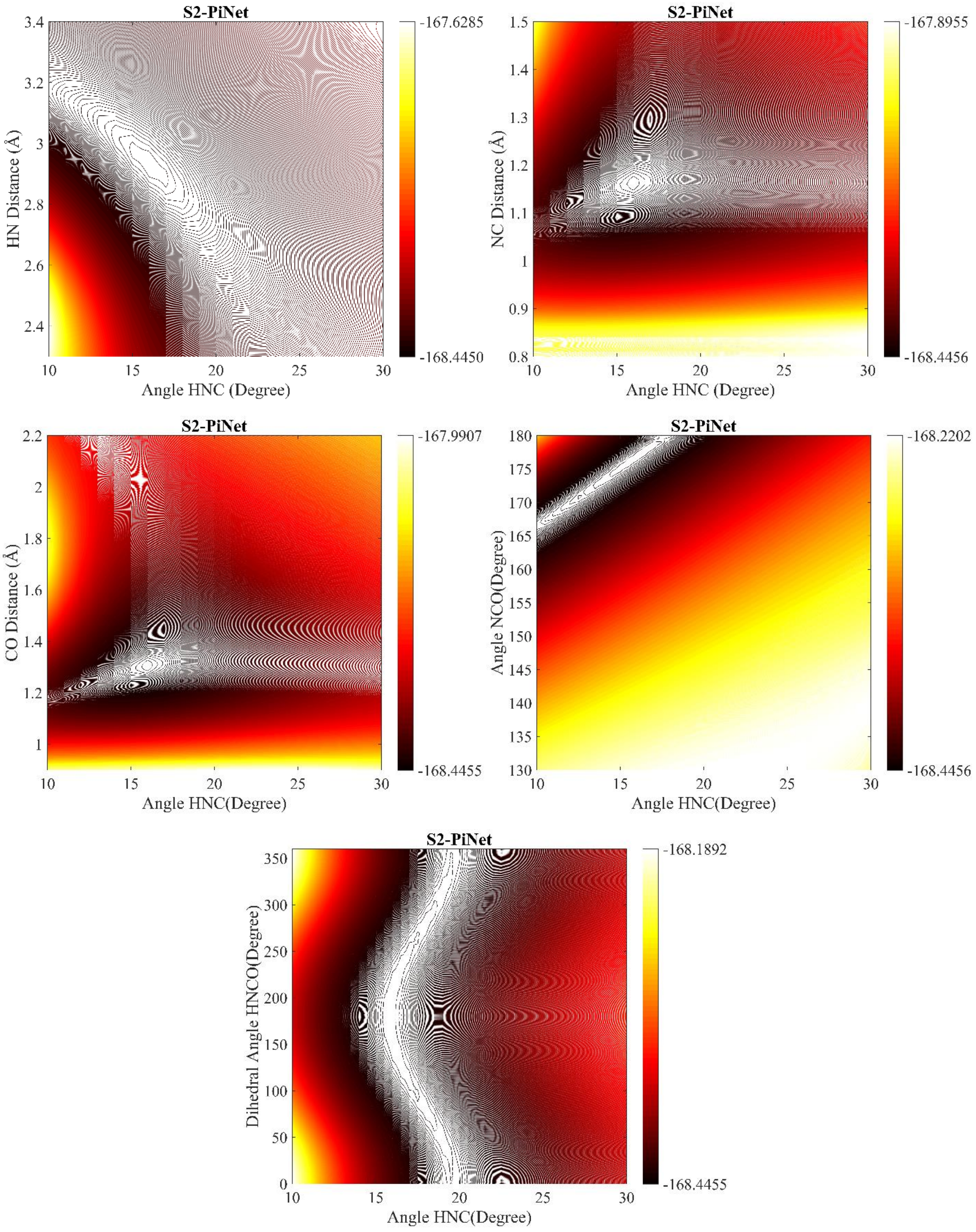

Figure S10. S2-PiNet reproduced contours. Energy unite is Hartree.

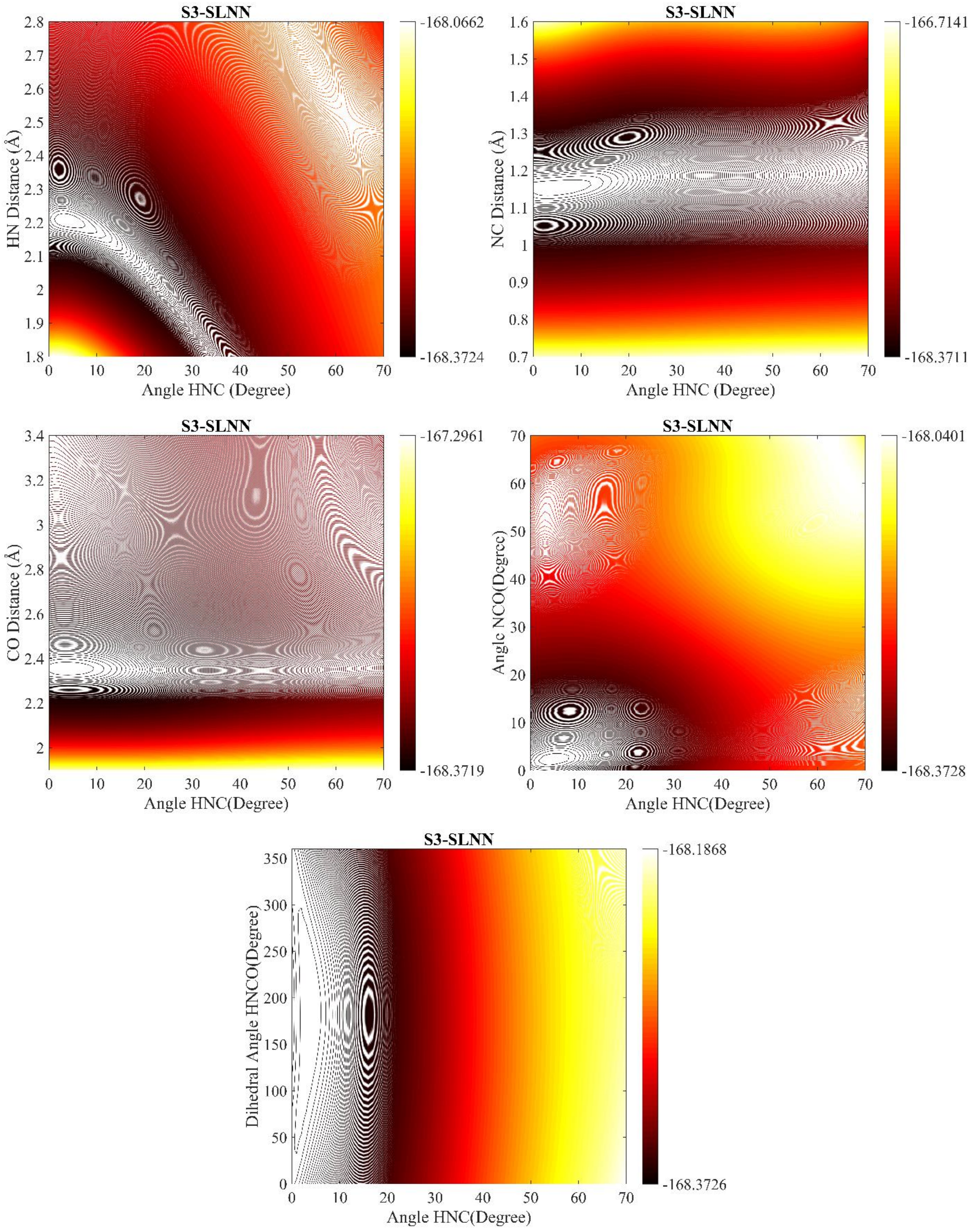

Figure S11. S3-SLNN reproduced contours. Energy unite is Hartree.

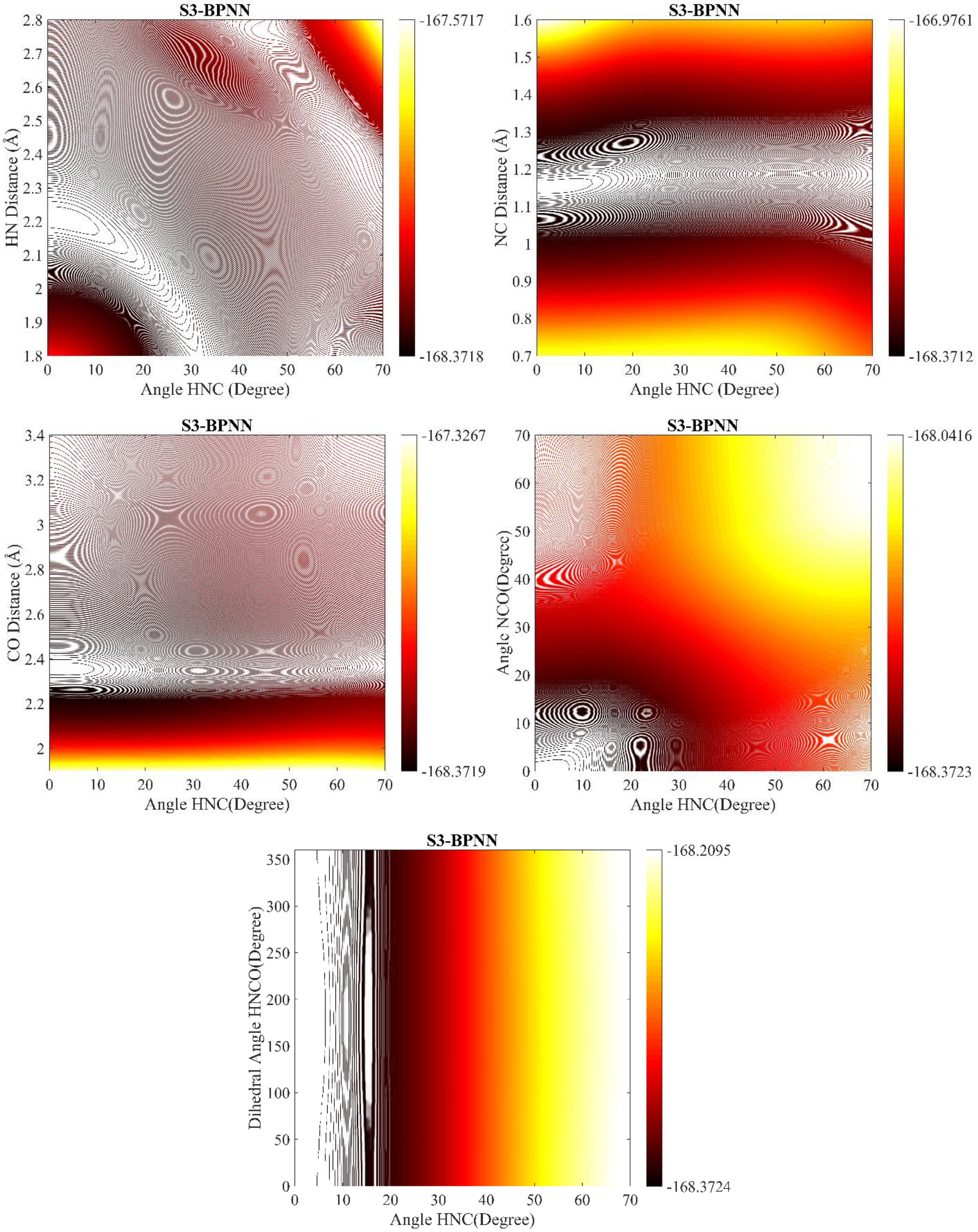

Figure S12. S3-BPNN reproduced contours. Energy unite is Hartree.

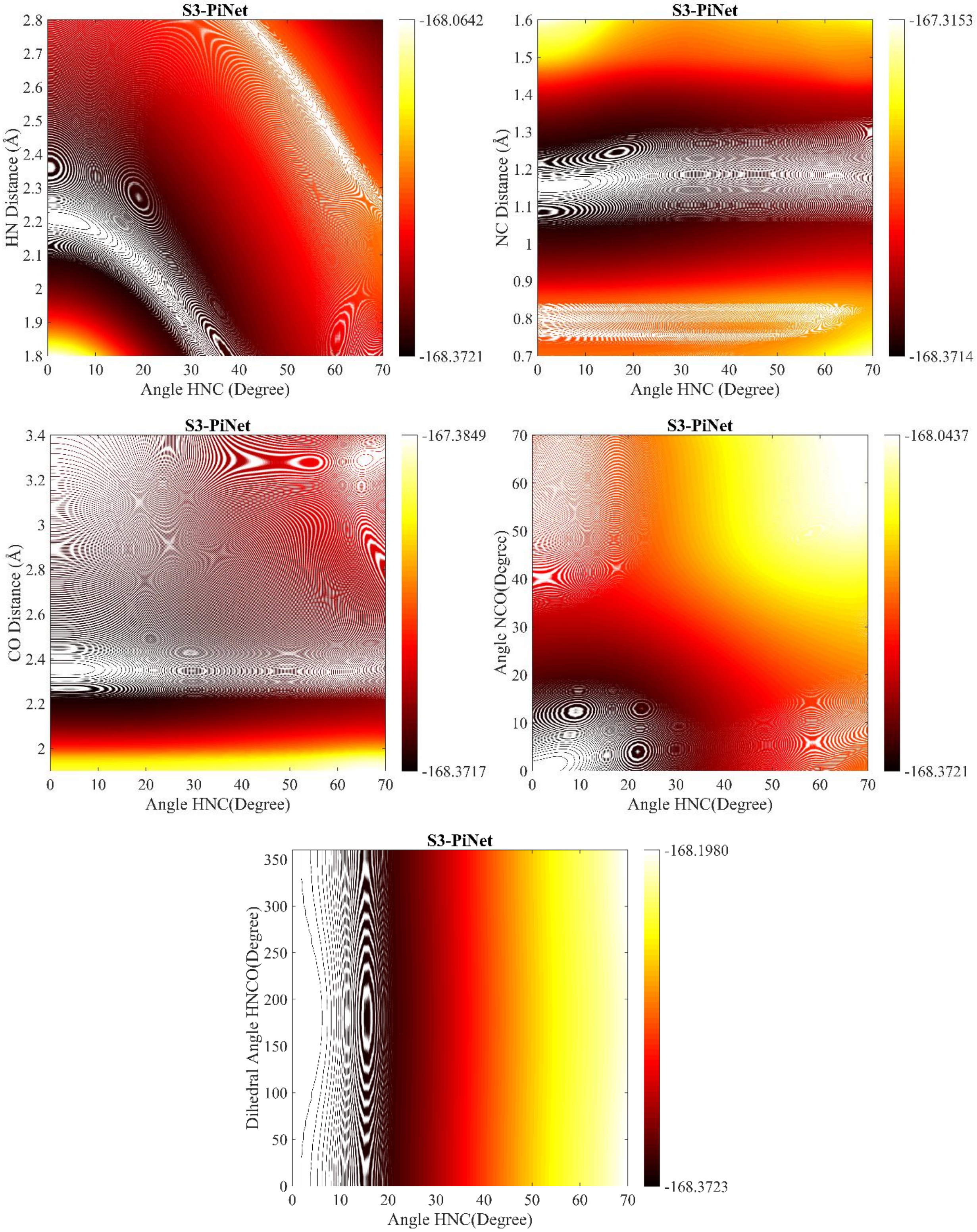

Figure S13. S3-PiNet reproduced contours. Energy unite is Hartree.

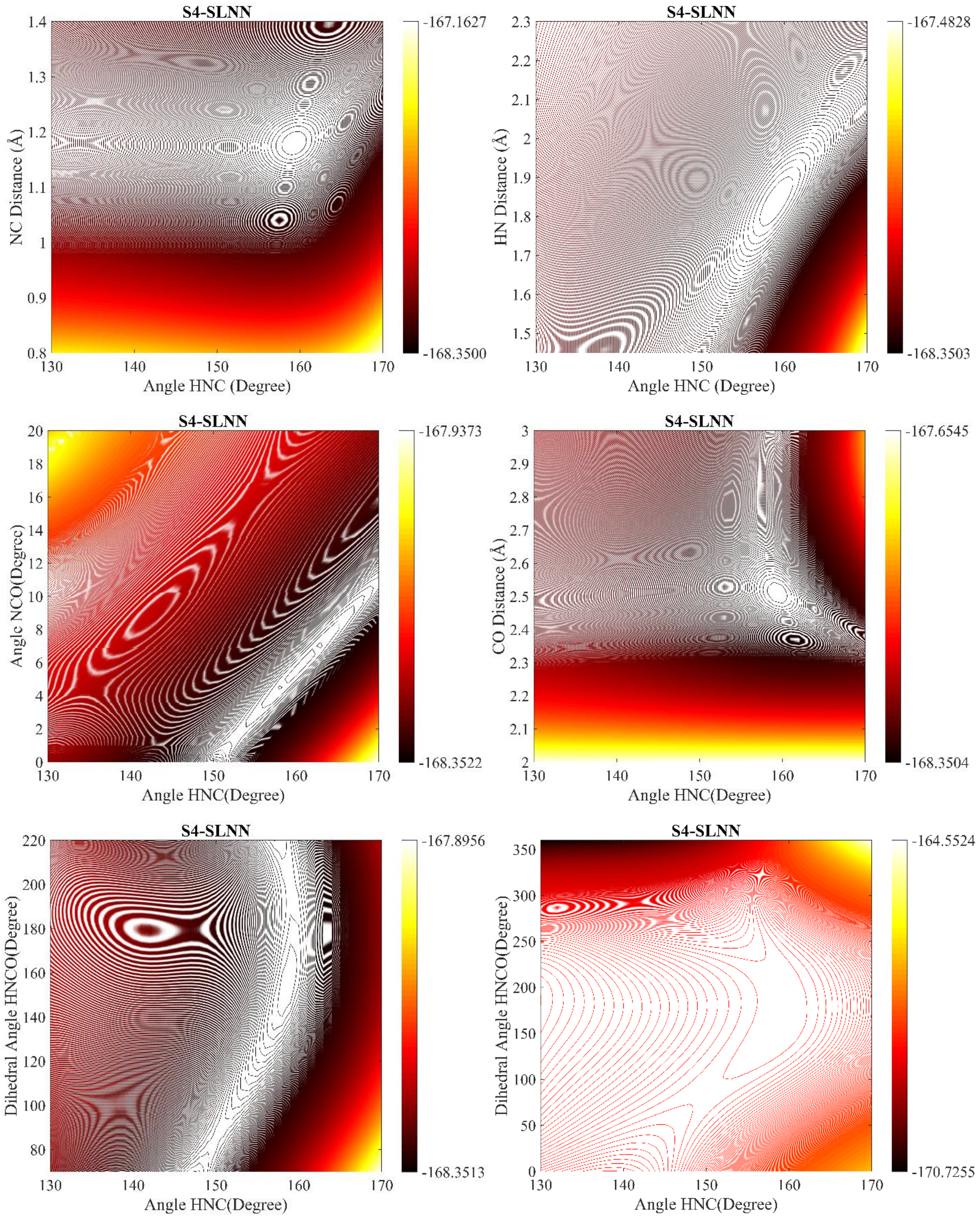

Figure S14. S4-SLNN reproduced contours. Energy unite is Hartree.

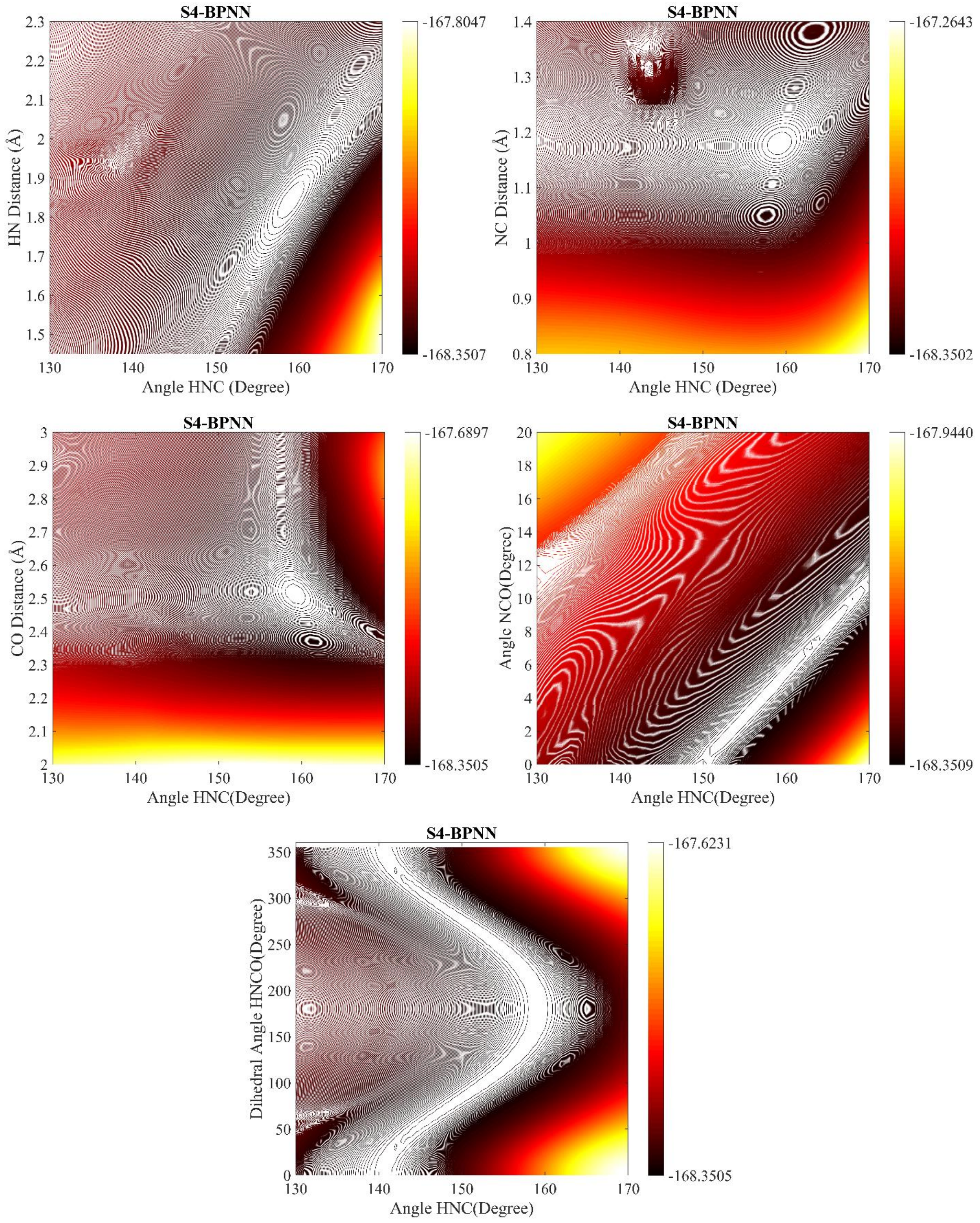

Figure S15. S4-BPNN reproduced contours. Energy unite is Hartree.

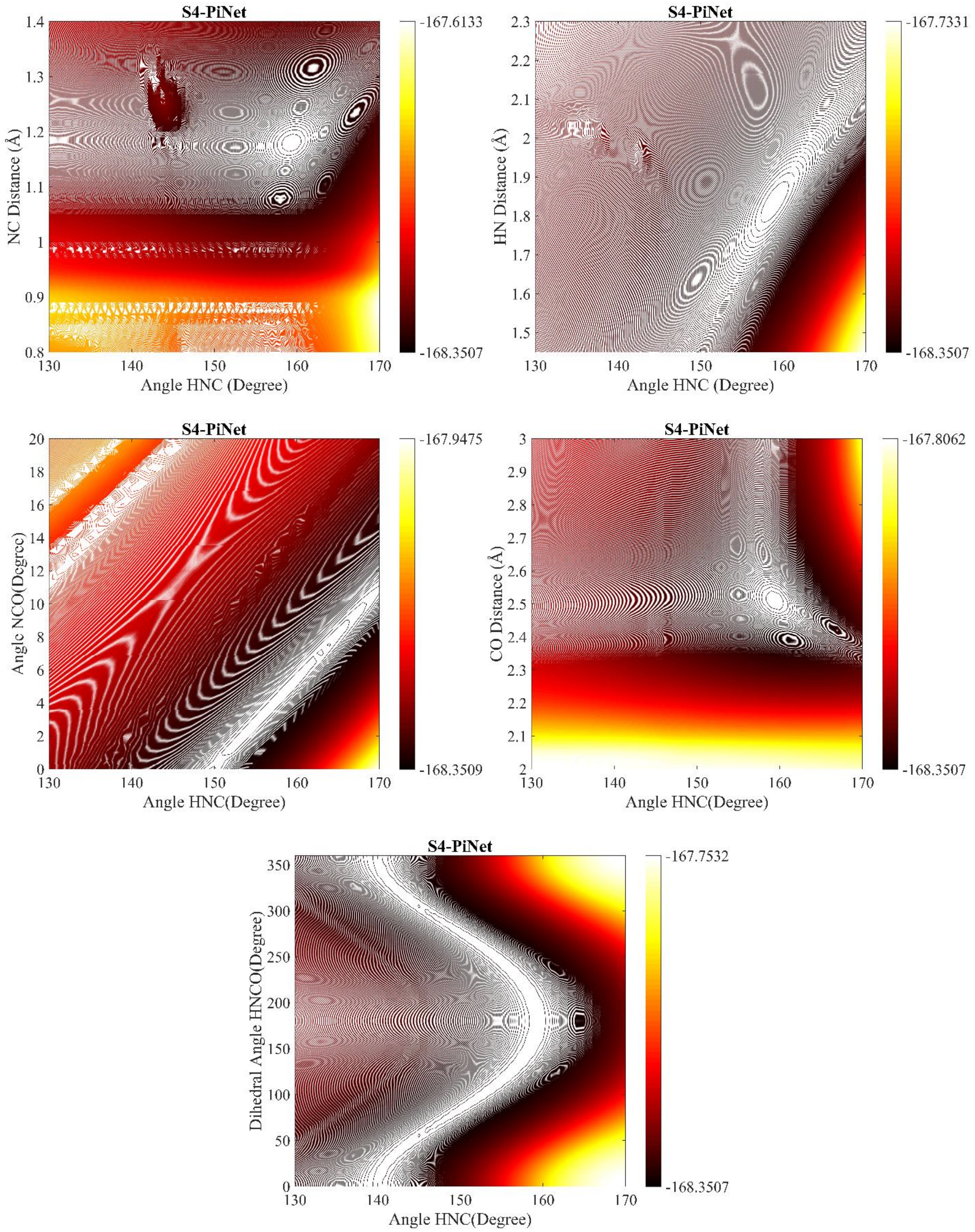

Figure S16. S4-PiNet reproduced contours. Energy unite is Hartree.

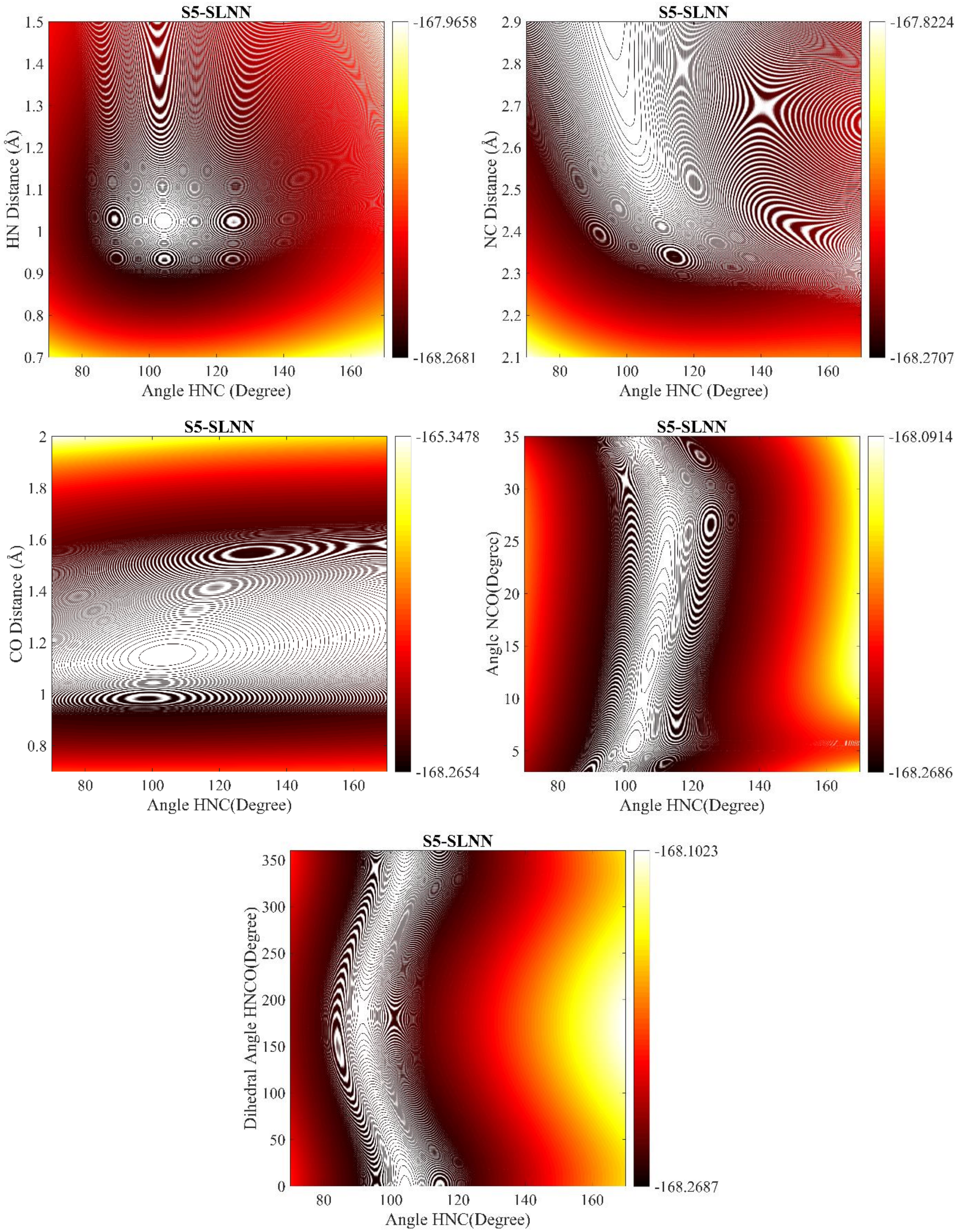

Figure S17. S5-SLNN reproduced contours. Energy unite is Hartree.

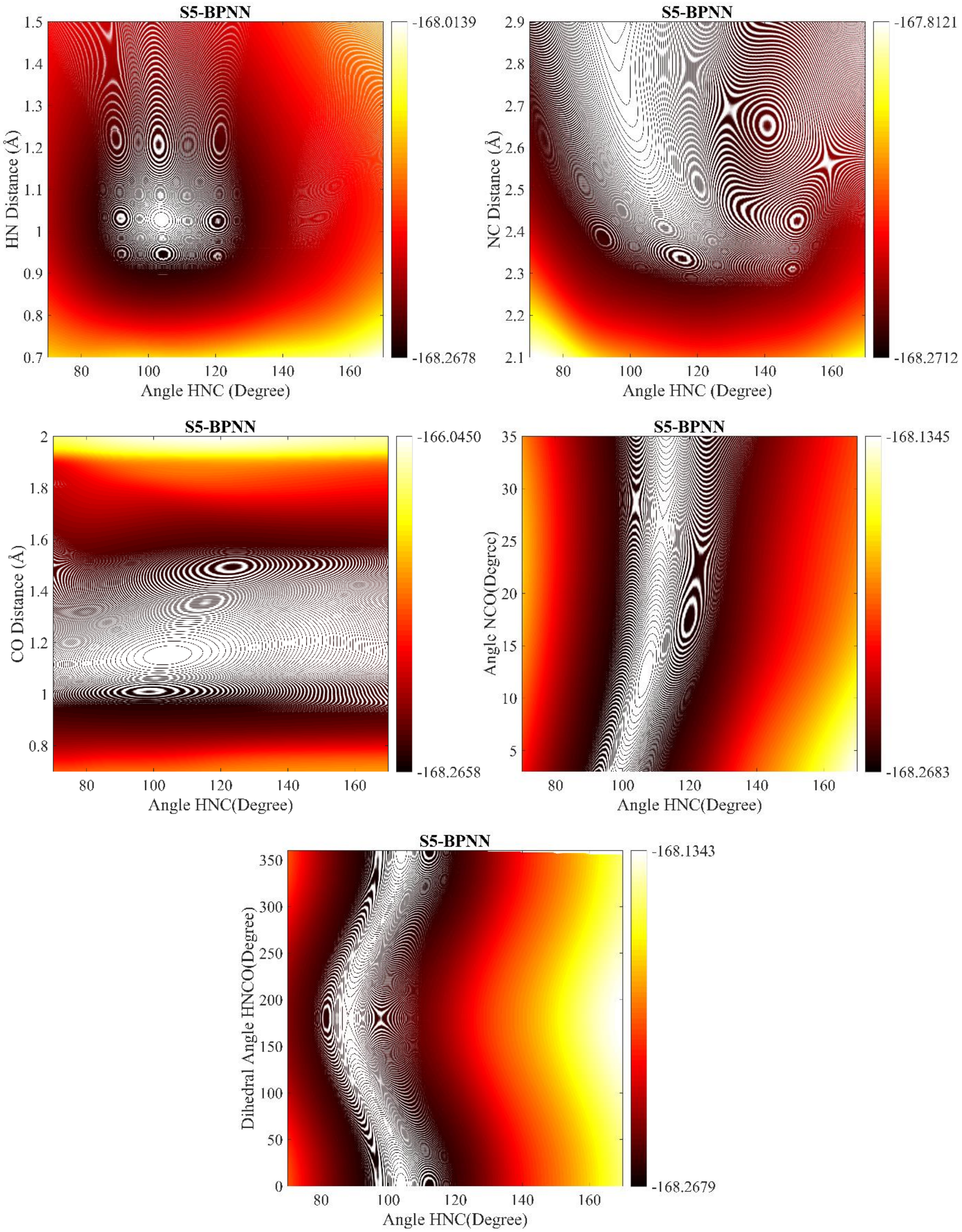

Figure S18. S5-BPNN reproduced contours. Energy unite is Hartree.

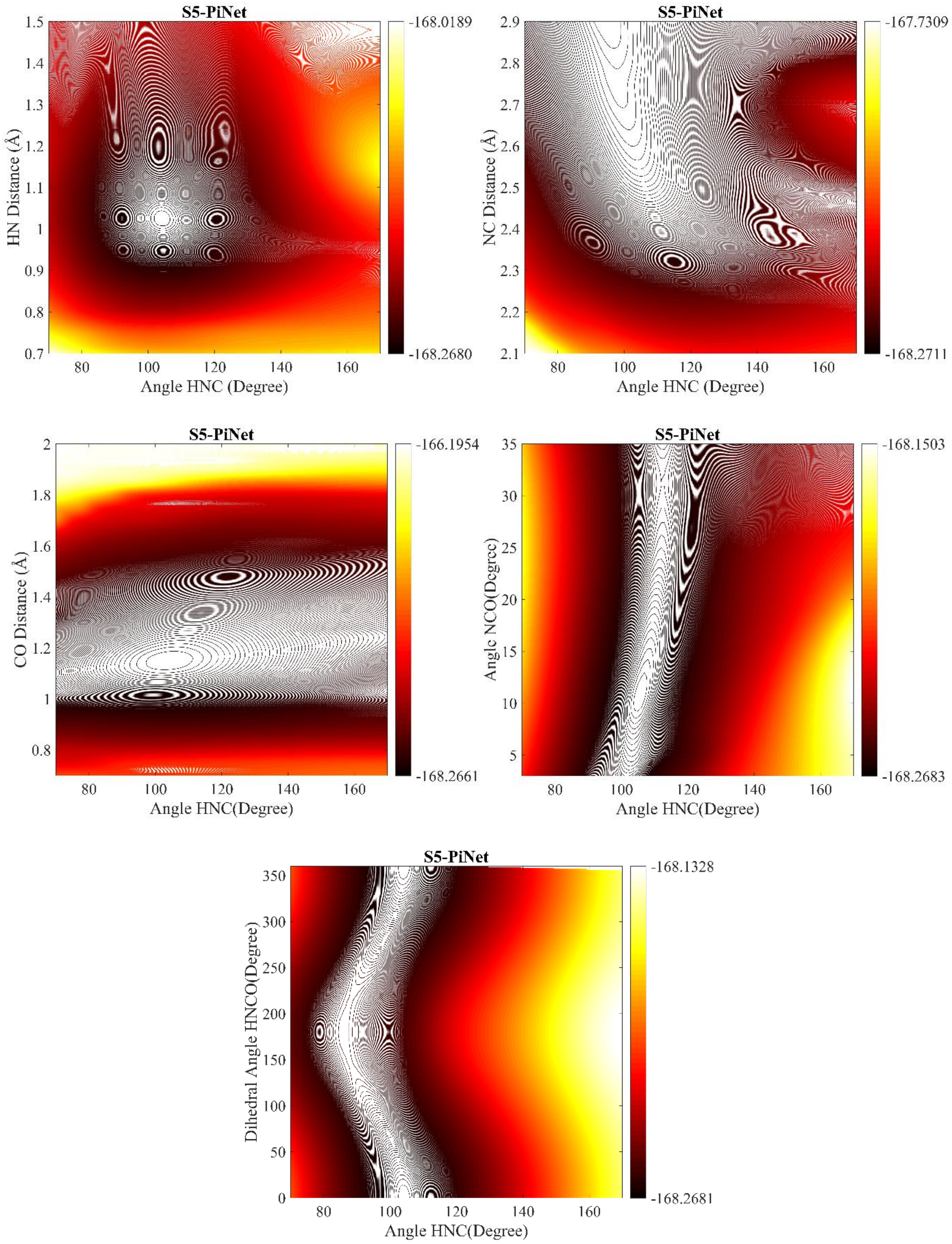

Figure S19. S5-PiNet reproduced contours. Energy unite is Hartree.

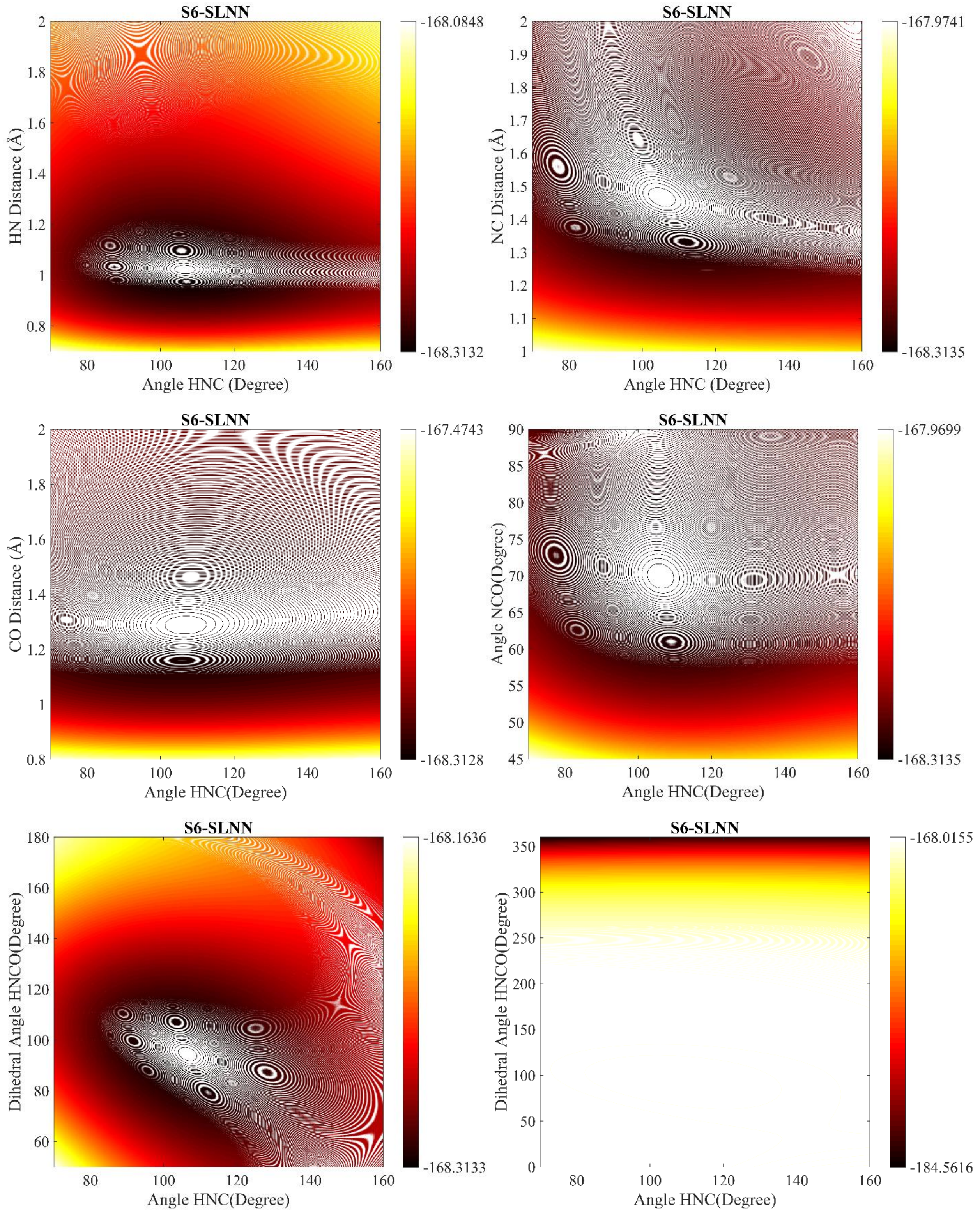

Figure S20. S6-SLNN reproduced contours. Energy unite is Hartree.

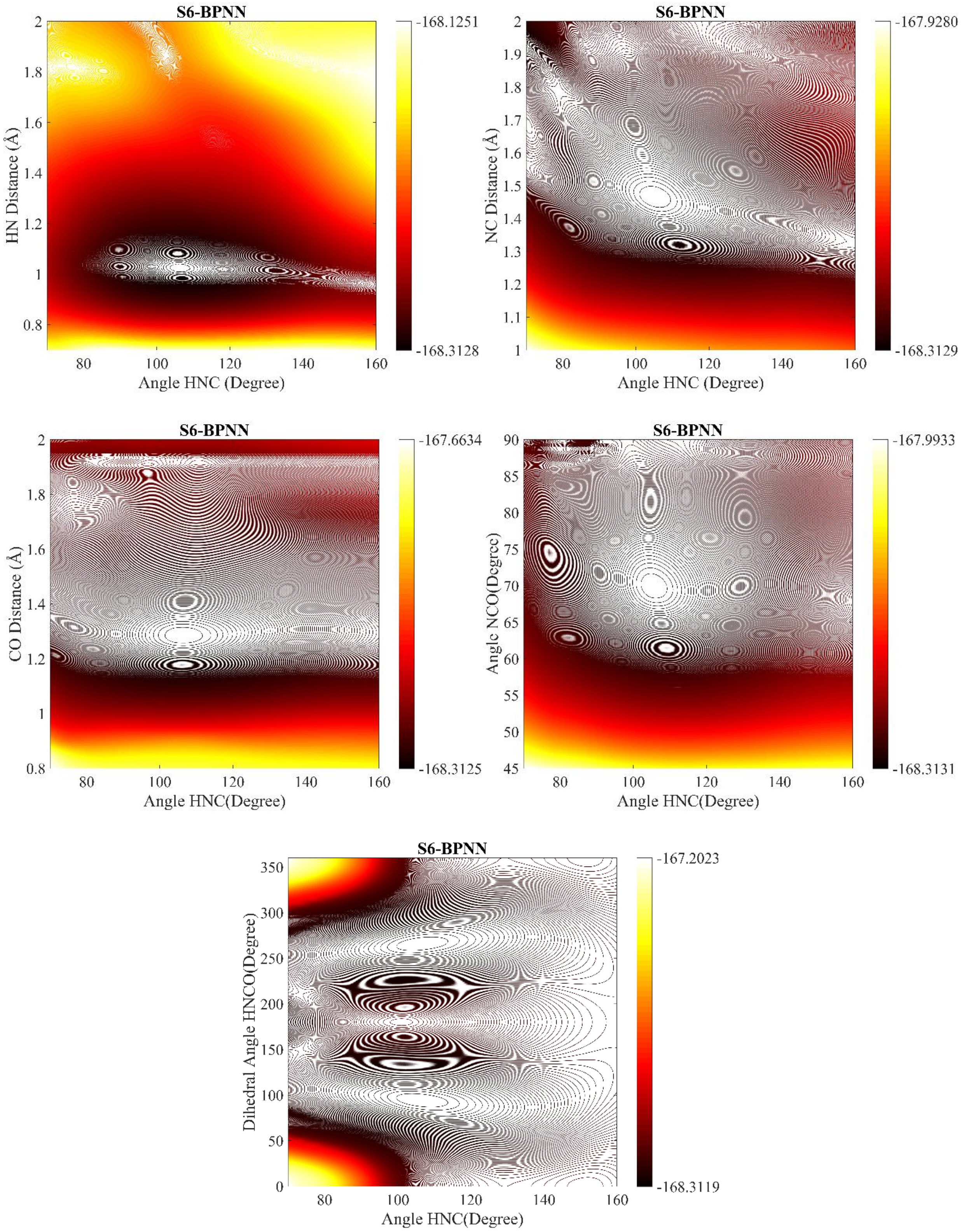

Figure S21. S6-BPNN reproduced contours. Energy unite is Hartree.

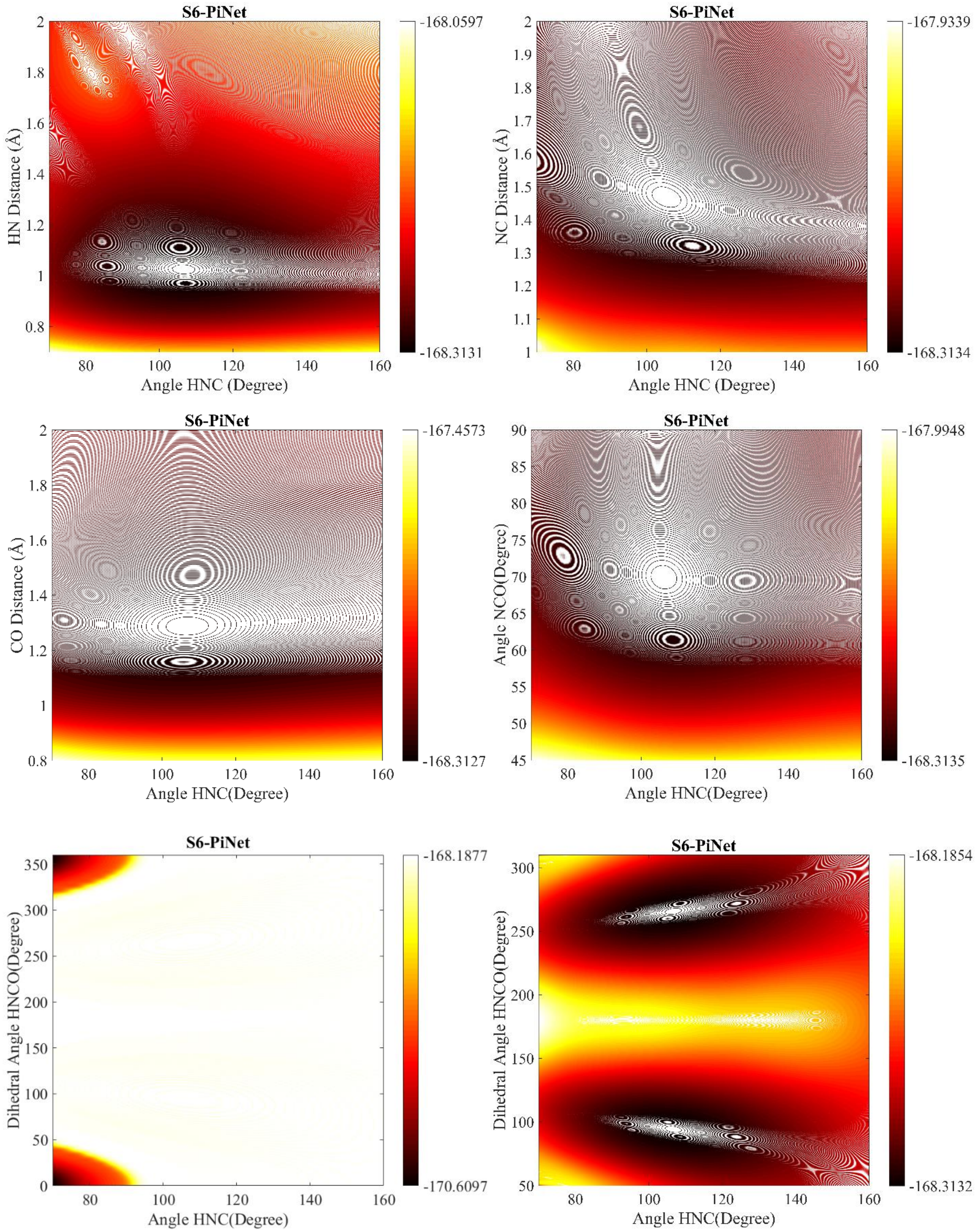

Figure S22. S6-PiNet reproduced contours. Energy unite is Hartre

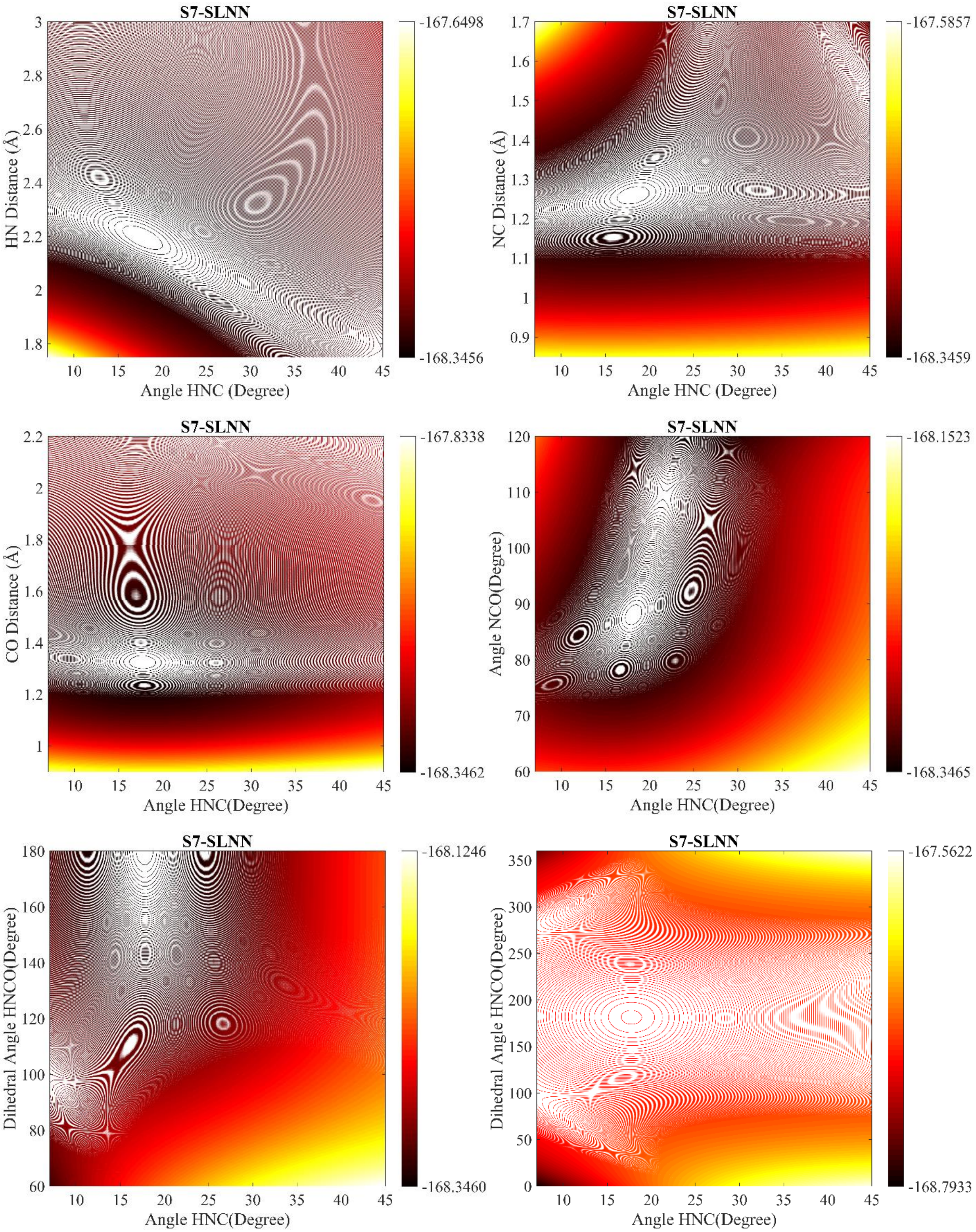

Figure S23. S7-SLNN reproduced contours. Energy unite is Hartree.

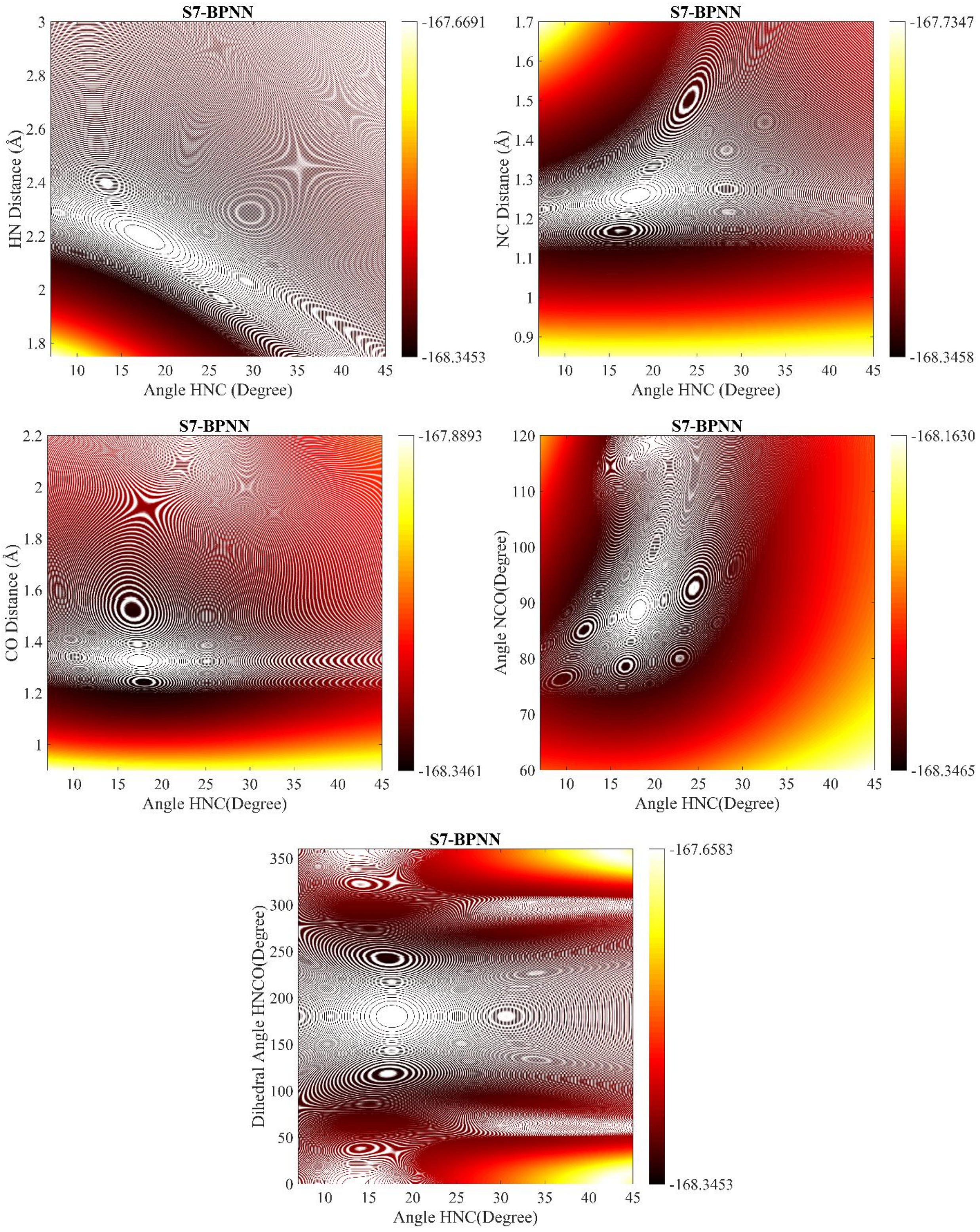

Figure S24. S7-BPNN reproduced contours. Energy unite is Hartree.

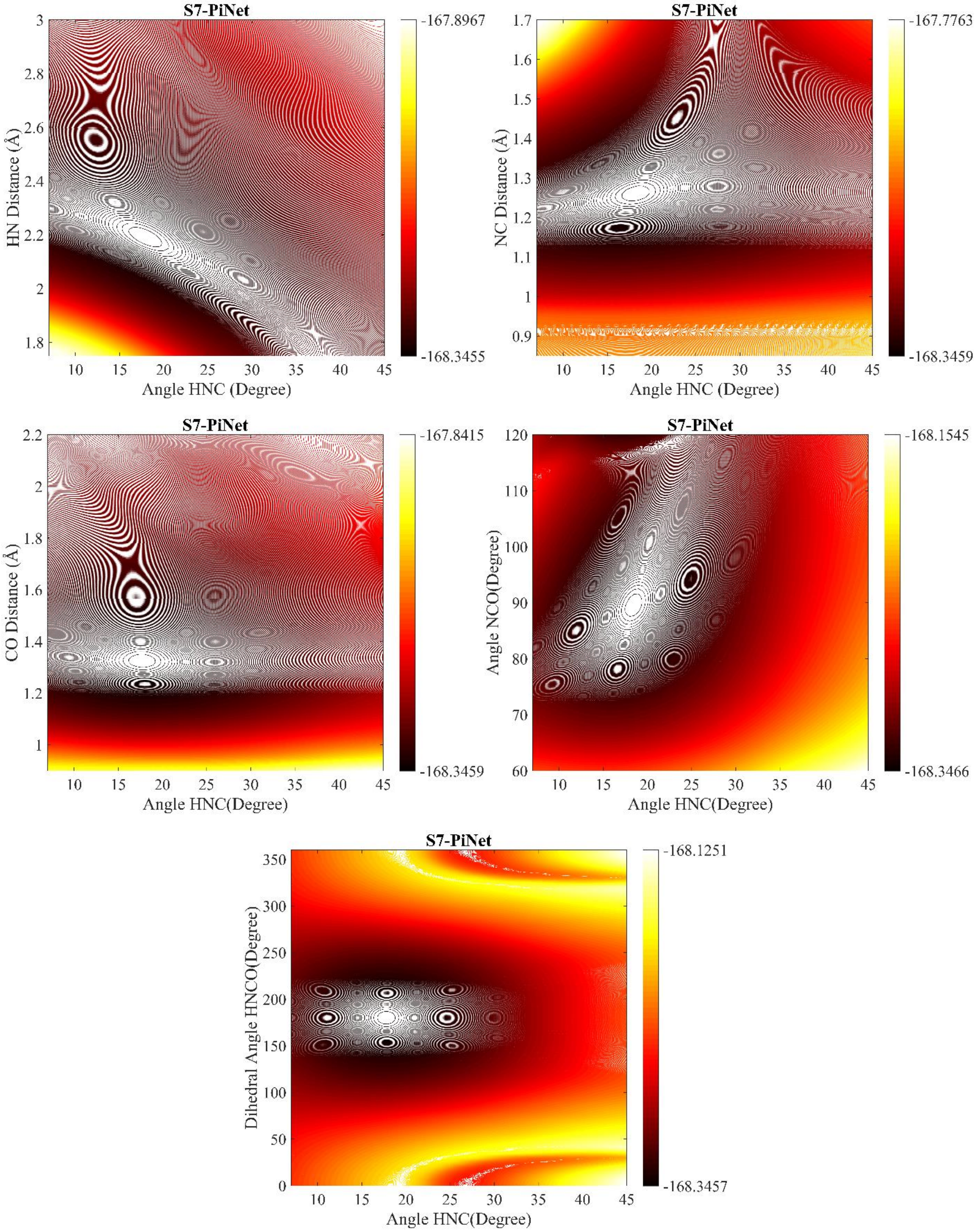

Figure S25. S7-PiNet reproduced contours. Energy unite is Hartree.

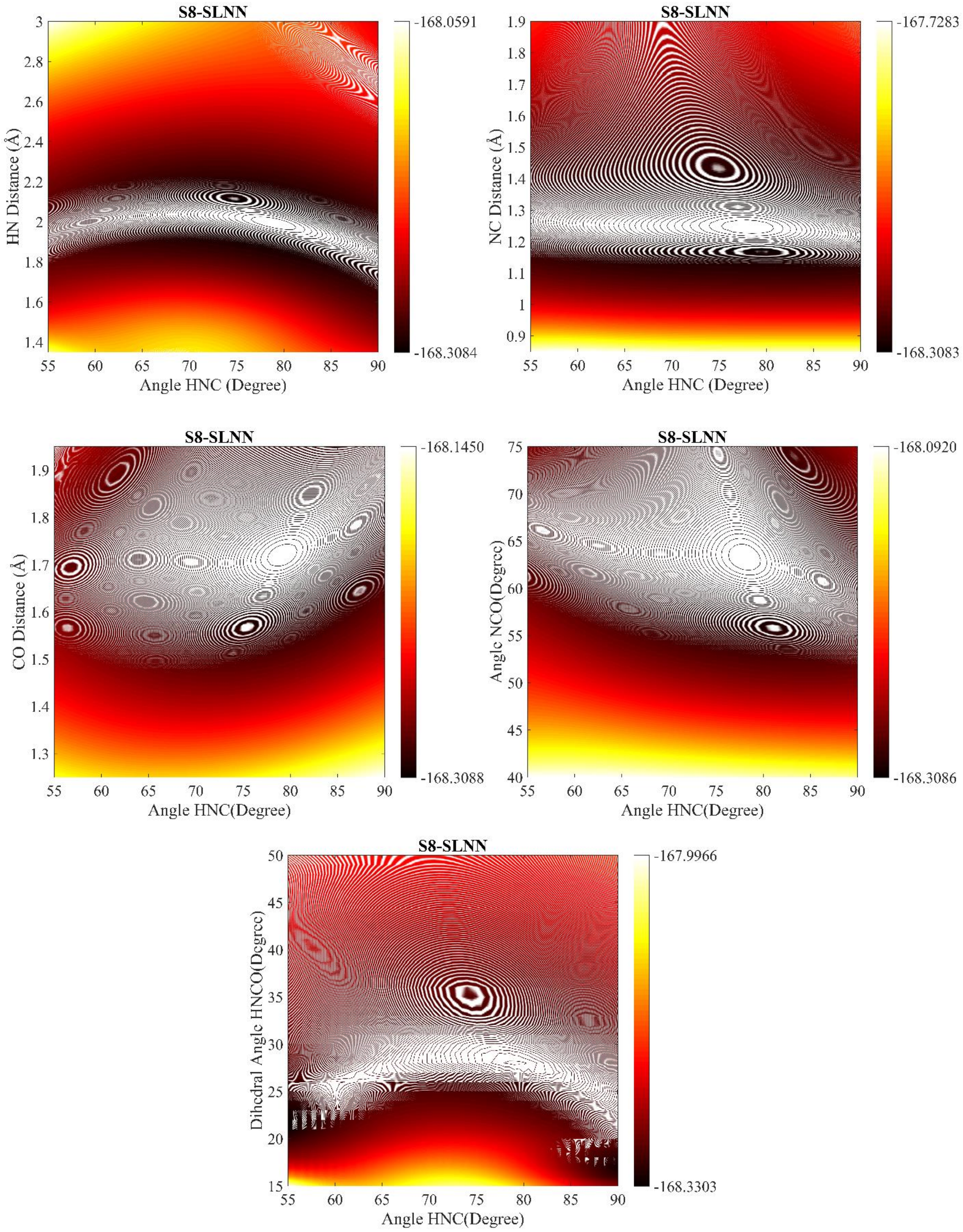

Figure S26. S8-SLNN reproduced contours. Energy unite is Hartree.

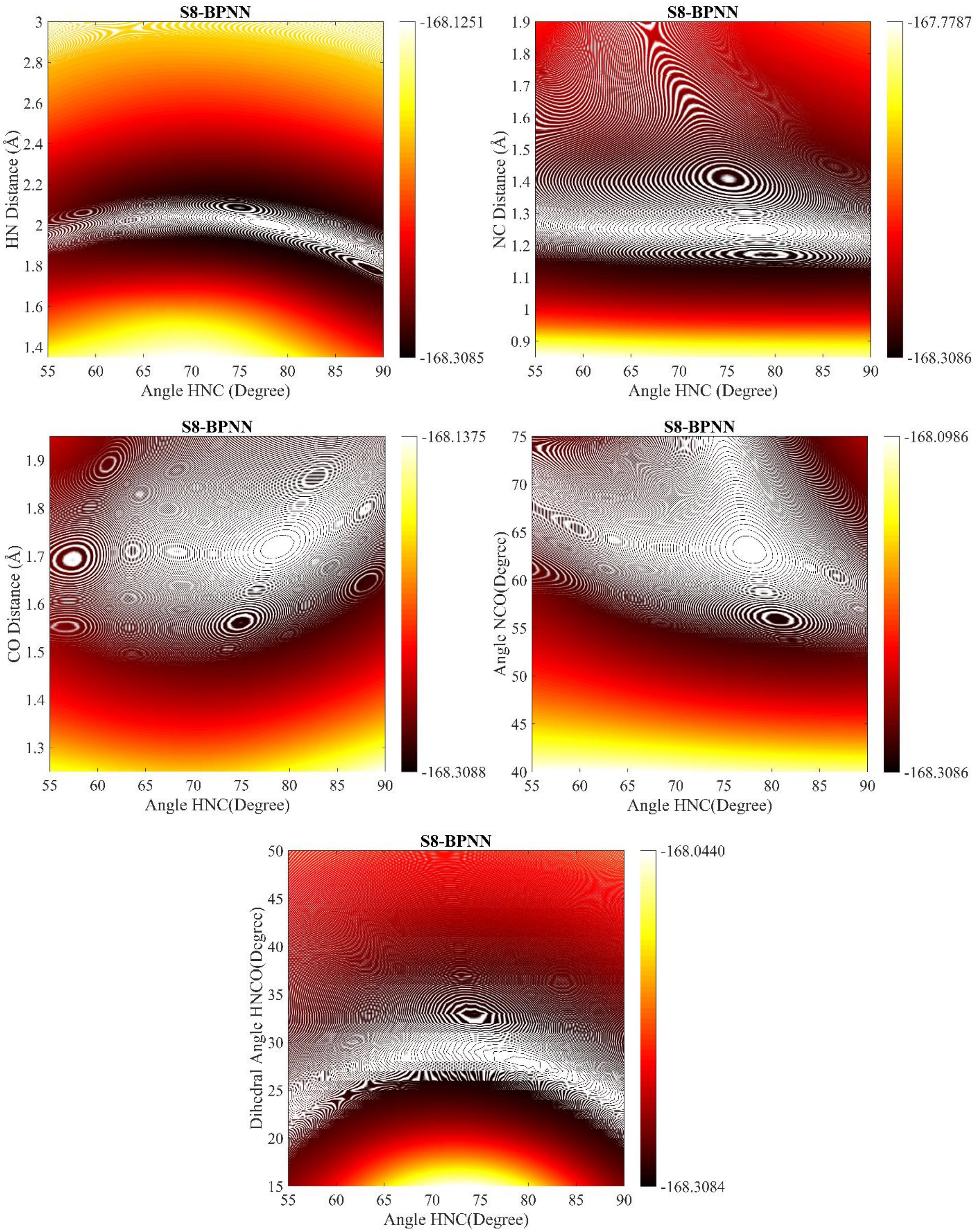

Figure S27. S8-BPNN reproduced contours. Energy unite is Hartree.

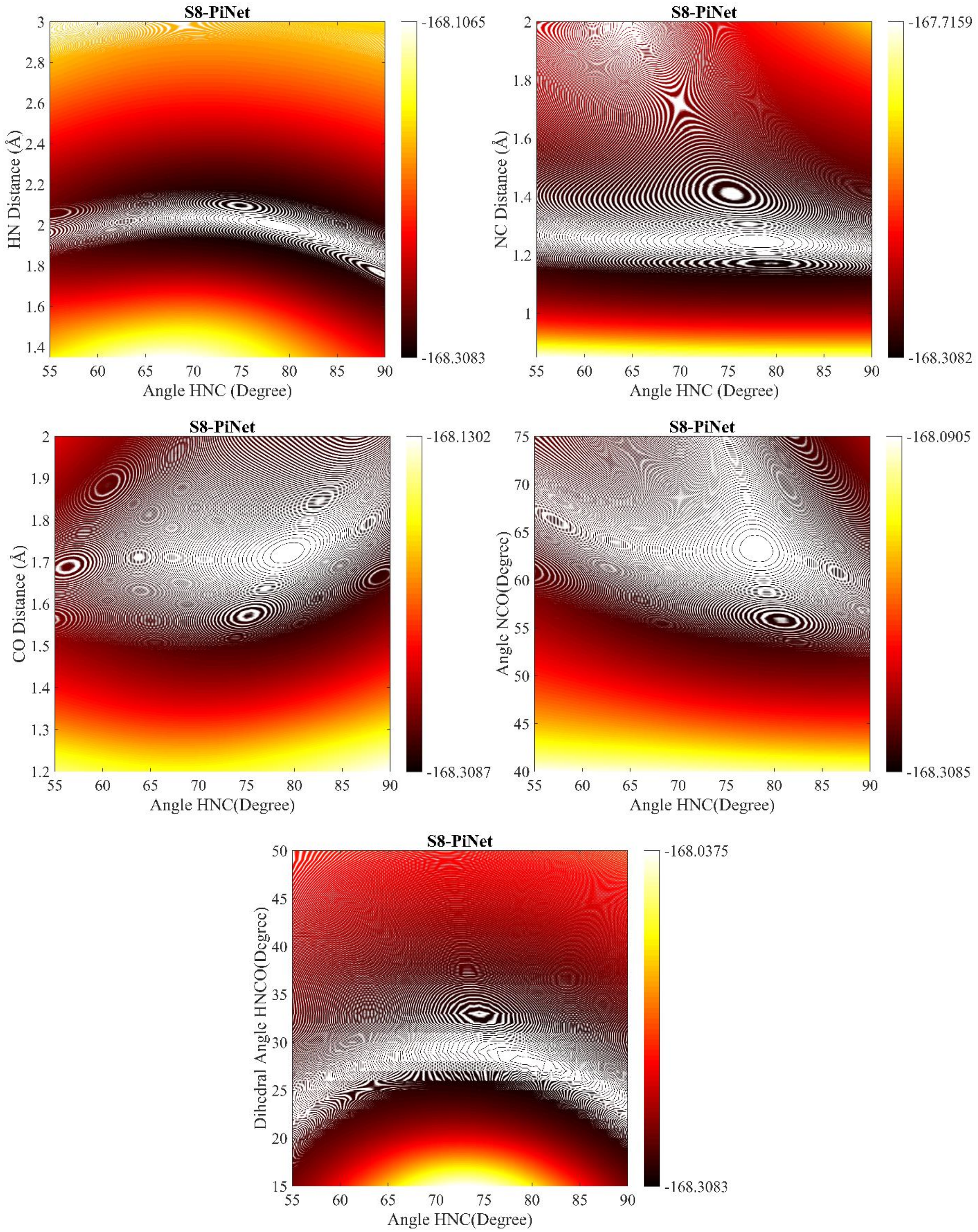

Figure S28. S8-PiNet reproduced contours. Energy unite is Hartree.

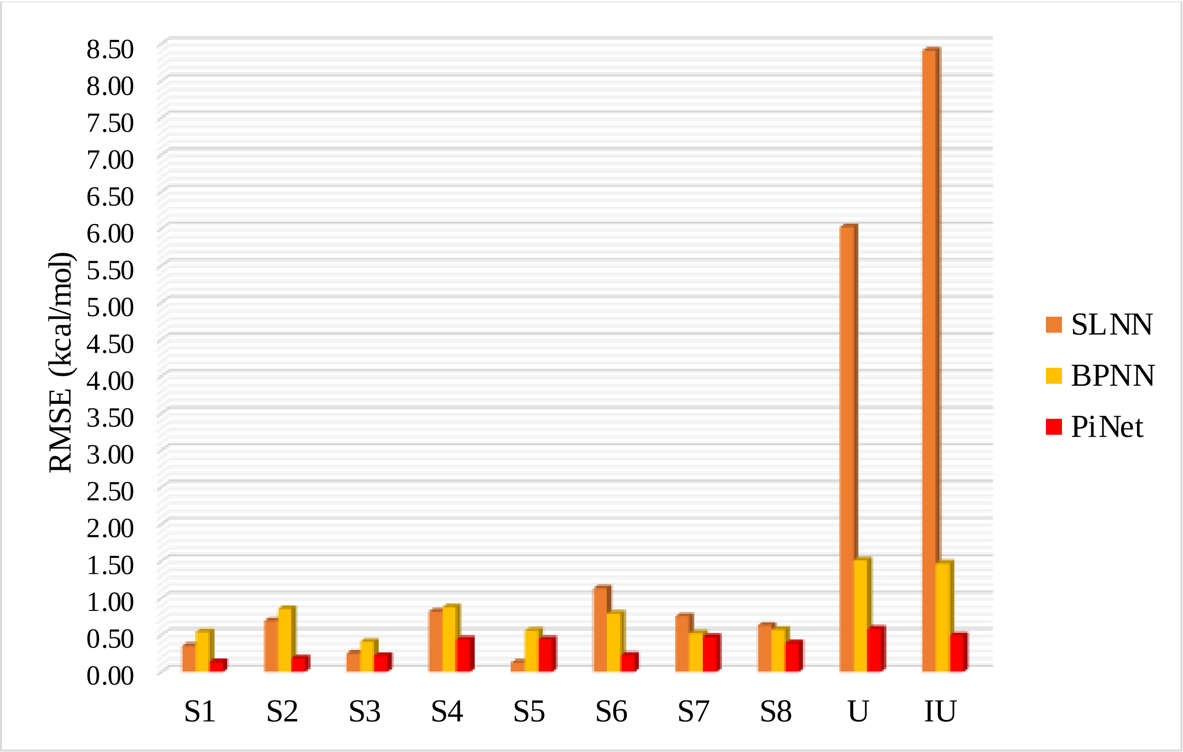

Figure S29. Comparison of the RMSEs for Sn-SLNN, Sn-BPNN, Sn-PiNet, U(IU)-SLNN, U(IU)-BPNN and U(IU)-PiNet networks.
